# Accuracy and precision analyses of single-time-point dosimetry utilising physiologically-based pharmacokinetic modelling and non-linear mixed-effects modelling

**DOI:** 10.1186/s40658-025-00726-7

**Published:** 2025-03-26

**Authors:** Indra Budiansah, Deni Hardiansyah, Ade Riana, Supriyanto Ardjo Pawiro, Ambros J. Beer, Gerhard Glatting

**Affiliations:** 1 Medical Radiation Physics, Department of Nuclear Medicine, Ulm University, Ulm, Germany; 2 Faculty of Mathematics and Natural Sciences, Medical Physics and Biophysics Division, Physics Department, Universitas Indonesia, Depok, Indonesia; 3 Radiation Protection and Compliance Testing Laboratory, Medical Devices and Facilities Safety Centre, Jakarta, Indonesia; 4 Department of Nuclear Medicine, Ulm University, Ulm, Germany

**Keywords:** PBPK, NLMEM, STP, Accuracy, Precision

## Abstract

**Purpose:**

The aim of this study was to investigate the accuracy and precision of single-time-point (STP) dosimetry using a physiologically-based pharmacokinetic (PBPK) model with non-linear mixed-effects modelling (NLMEM).

**Methods:**

Biokinetic data of [^111^In]In-DOTA-TATE in tumours, kidneys, liver, spleen, and whole body were collected from eight patients. The imaging was performed using planar scintigraphy at 2, 4, 24, 48, and 72 h after injection. Serum activity concentration was quantified at 5 and 15 min; 0.5, 1, 2, and 4 h; and 1, 2, and 3 d after injection. The PBPK model was fitted to the biokinetic data using NONMEM software version 7.5.1. Goodness-of-fit (GoF) criteria were visual inspection of the biokinetic curves, relative standard errors (RSEs) of the fitted parameters < 50%, and the absolute values of the off-diagonal elements in the correlation matrix < 0.8. All-time-point (ATP) fitting was performed, and the obtained absorbed doses (ADs) were used as reference (rADs). The leave-one-out Jackknife method was applied to calculate STP ADs (sADs). The accuracy of STP dosimetry was evaluated using the relative deviation between sADs and rADs. The time point, which resulted in the smallest root-mean-square error (RMSE), was selected as the optimal time point for STP dosimetry. The precision of the AD was calculated as ratio of AD RSE and AD values.

**Results:**

The ATP fitting was adequate based on the GoF test. STP dosimetry at 48 h after injection provided an acceptable estimation of ADs, yielding the lowest RMSE values for the kidney and tumour, calculated as (7 ± 2)% and (14 ± 4)%, respectively. The ADs in STP dosimetry showed lower precision than in ATP dosimetry. For instance, the ADs precision in ATP and STP dosimetry for kidneys in term median[min, max] were 3[3, 3]% and 6[5, 6]%, respectively. Similar results were found for the tumours where the precision of the ADs in ATP and STP dosimetry were 4[4, 5]% and 9[8, 12] %, respectively.

**Conclusion:**

STP dosimetry exhibits acceptable accuracy, although it shows a decrease in precision compared to ATP fitting. Precision information is clinically relevant for developing the optimal strategies for simplified dosimetry protocols.

**Supplementary Information:**

The online version contains supplementary material available at 10.1186/s40658-025-00726-7.

## Background

In molecular radiotherapy (MRT), the accurate calculation of the individual time-integrated activity coefficients (TIACs), used to calculate the absorbed dose (AD), is desirable for optimising the safety and efficacy of treatment [1–3]. Using single-time-point (STP) biokinetic data for TIAC calculation has gained popularity due to its lower workload and reduced patient burden compared to conventional TIAC calculation methods that require multiple-time-point biokinetic measurements [4–7].

Among various approaches for calculating TIACs, physiologically-based pharmacokinetic (PBPK) modelling has been shown to be a promising method [2, 8]. Such models simulate the behaviour of radiolabelled substances based on key physiological processes—administration, distribution, metabolism, and excretion (ADME). PBPK models offer significant advantages, including accurate selection of the administered activity, drug interaction simulations, and concentration–time profiles across multiple organs [9]. Furthermore, PBPK models integrate existing knowledge of human physiology and drug-specific parameters, enabling optimised simultaneous simulations of radiopharmaceuticals' ADME across different organs. Recent studies have demonstrated that integrating PBPK modelling with Non-Linear Mixed-Effects Modelling (NLMEM) for STP dosimetry can yield accurate estimates of TIAC in various organs simultaneously [10].

The precision of individual TIACs in MRT is as important as their accuracy. An example highlighting the importance of incorporating TIAC precision in MRT is observed in multi-cycle therapies. Current dosimetry protocols recommend estimating TIACs using multiple-time-point biokinetic data for the first cycle, followed by STP biokinetic data for subsequent cycles [11–13]. Assessing the TIAC profiles between treatment cycles may be suboptimal without including precision information on individual TIACs in each cycle. Furthermore, recent European Association of Nuclear Medicine guidelines recommend incorporating individual TIAC’s precision (or uncertainty) into AD calculations [14]. Incorporating precision information, such as the standard error of individual TIACs, allows optimisation of therapy dosimetry in different cycles. Precision information can be used to assess whether the AD remains consistent or varies between cycles. This information provides a basis for determining whether adjustments to the administered activity in subsequent cycles are necessary. However, there is a notable gap in the literature regarding the precision analysis of the individual TIAC estimation in STP dosimetry utilising the PBPK model. Understanding the precision in individual TIAC calculations is crucial, yet it is underexplored in MRT. Individual TIAC’s precision information is desirable for quality assurance and quality control.

Most studies on STP TIAC calculations in the literature focus on accuracy [4, 15], with limited research investigating precision [16, 17]. Although the accuracy information of STP dosimetry is beneficial for testing the validity of the methods, precision information is also desirable to judge the reliability of the results. This study proposes applying a PBPK model to calculate TIAC precision, as it has been demonstrated to be a good modelling approach for the biokinetics of radiopharmaceuticals [8, 9, 18]. A PBPK model analyses all organ data simultaneously, which could improve the fitting process in the TIAC estimation while enhancing the fitting outcome. Thus, a method to calculate and quantify both the accuracy and precision of individual TIACs from STP dosimetry using a PBPK model and NLMEM is desirable. In this study, we present a general method to calculate the accuracy and precision of individual TIAC in STP dosimetry using a PBPK model and NLMEM in various organs, demonstrated in an example of [^90^Y]Y-DOTA-TATE therapy. This study was conducted based on the work by Hardiansyah et al. [10], with several improvements: (1) the precision analysis of individual ADs, which were not calculated in the previous study, has been incorporated to enhance the reliability of the dosimetry evaluation in this study; and (2) the set of the fitted PBPK parameters has been refined and improved based on the model selection analysis to capture the biokinetic data better, addressing the limitation of non-optimal parameter setting in the earlier study.

## Materials and methods

### Biokinetic data

Biokinetic data was collected from a group of eight patients, consisting of four with NETs and four with meningiomas. All patients were scheduled for two to three cycles of peptide receptor radionuclide therapy utilising [^90^Y]Y-DOTA-TATE [19]. The radiopharmaceutical was administered intravenously within (51 ± 8) min. The amount of administered activity was (75 ± 10) nmol DOTA-TATE labelled with (140 ± 14) MBq ^111^In. Arginine and Lysine (1,000 mL, 2.5% infusion) were administered over a 2 h period, commencing 0.5 h before administering [^111^In]In-DOTA-TATE. Pre-treatment planar whole-body scintigraphies were performed at 2, 4, 24, 48, and 72 h after injection using a double-head gamma camera (ECAM, Siemens, Erlangen, Germany) [19]. A medium energy collimator with energy windows A1 = 171 keV (width 15%), A2 = 245 keV (15%), B1 = 142 keV (18%), and B2 = 205 keV (18%) was used. The images were adjusted for scatter based on the formula ${\text{Isc}} = \left[ {A1 + A2} \right] - k \times \left( {B1 + B2} \right)$, where $k$ is the scatter factor with a value of 0.6 [20]. This study utilised the amount of the radiopharmaceutical in blood serum, whole body, kidneys, spleen, liver, and tumours [19]. The measurement of organ activity as a function of time was adjusted for background correction and self-attenuation, following the guidelines outlined in the MIRD pamphlet number 16 [21]. The concentration of blood serum activity was quantified using a gamma counter (auto-gamma-5003; Canberra Packard) at 5 and 15 min; 0.5, 1, 2, and 4 h; and 1, 2, and 3 d after injection. The CT scan was used to assess the volumes of the liver, kidneys, spleen, and tumour lesions. The regions of interest (ROIs) of all visible tumour lesions in planar images were drawn, and the two largest tumours were delineated and quantified [19]. The sum of the biokinetic data from this quantification of the two largest tumours was used in this study. The segmentation of the organs and tumours in the planar images was performed by an experienced medical physicist in close consultation with a medical doctor [19]. The biokinetic data of [^111^In]In-DOTA-TATE was used as a surrogate for the biokinetics of [⁹⁰Y]Y-DOTA-TATE in peptide receptor radionuclide therapy, as recommended in the literature [19, 22].

### Physiologically-Based Pharmacokinetic Structural Model

**Fig. 1:**
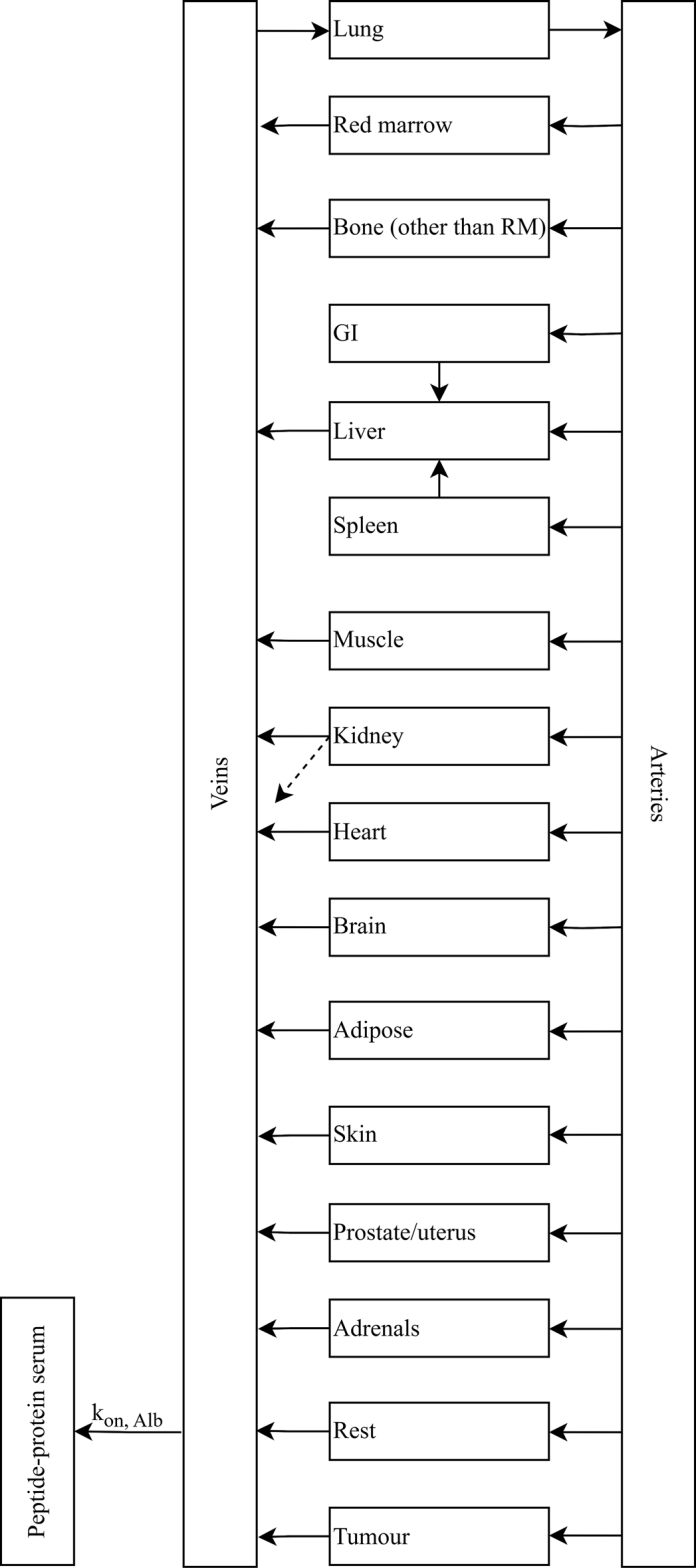
PBPK structural model adapted from literature [19]. The model consists of a series of rectangles representing organs connected by blood flow, as shown by the arrows

This study used a comprehensive whole-body PBPK model that was specifically built for treatment planning in peptide receptor radionuclide therapy [19]. The model describes the process of radiopharmaceutical distribution through blood circulation, transfer to the organ, specific binding to the target site, internalisation, and the clearance from the organs. Figure 1 describes the PBPK structural model, and the physiological parameters are in the supplemental file, Table S1. The densities of somatostatin receptors in the kidneys $\left( {\left[ {R_{{{\text{k}},0}} } \right]} \right)$, liver $\left( {\left[ {R_{{{\text{L}},0}} } \right]} \right)$, spleen $\left( {\left[ {R_{{{\text{S}},0}} } \right] } \right)$, tumours $\left( {\left[ {R_{{{\text{TU}},0}} } \right]} \right)$, and rest of the body $\left( {\left[ {R_{{{\text{Rest}},{ }0}} } \right]} \right)$, the degradation rates from tumours $\left( {\lambda_{{{\text{deg}}, {\text{TU}}}} } \right)$ and normal tissue $\left( {\lambda_{{{\text{deg}},{\text{ NT}}}} } \right)$, internalization rates of tumour $\left( {\lambda_{{{\text{int}},{\text{ TU}}}} } \right)$ and normal tissue $\left( {\lambda_{{{\text{int}},{\text{ NT}}}} } \right)$, and the binding rate to albumin in the blood $\left( {k_{{{\text{on}},{\text{ Alb}}}} } \right)$ were the fitted parameters of the PBPK model [19]. The parameters were estimated by fitting the PBPK model to the biokinetic data within the NLMEM. The values of all other parameters were fixed to be in line with those reported in the literature or patient-specific values [19].

### Non-linear mixed-effects modelling

In NLMEM, the individual parameters were defined as the combination of the fixed-effects (FE) and random-effects (RE) [23]. FE refers to the mean value of the parameters in the population, whereas RE (inter- and intra-individual variability) refers to the variation of the individual parameter from the mean value [24]. The exponential error model was used to explain the inter-individual variability since it ensures a positive parameter value, as recommended in the literature [23–25]. Thus, the individual parameter was modelled as follows:

1 $$P_{{{\text{ki}}}} = \theta_{{{\mu k}}} e^{{\eta_{ki} }} ; \,\eta_{{{\text{ki}}}} \sim N\left( {0,\sigma^{2} } \right)$$

where $P_{{{\text{ki}}}}$ is the parameter $k$ for individual $i$; $\theta_{\mu k}$ is the FE of the parameter $k$; and $\eta_{{{\text{ki}}}}$ is the RE of the parameter $k$ for individual $i$. The value of $\eta_{{{\text{ki}}}}$ is normally distributed with mean zero and variance value of $\sigma^{2}$. The difference between the model’s prediction and the observed data set was described in the intra-individual variability. The proportional error model was selected to elucidate the intra-individual variability [24, 26]. Therefore, the observed data was expressed as follows:

2 $$Y_{{{\text{obs}},i}} = Y_{{{\text{pred}},i}} \left( {1 + a \times \varepsilon } \right)\,\,\, ; \varepsilon \sim N\left( {0,1} \right)$$

where $Y_{{{\text{obs}},i}}$ is the observed data for individual $i$; $Y_{{{\text{pred}},i}}$ is the model’s prediction for individual $i$; $a$ is the error model parameter; and $\varepsilon$ is a random number that follows a normal distribution with a mean of zero and a variance of one. To assess the accuracy of the PBPK model's predictions for each tumour and investigated organs, the intra-individual variability was estimated in each tumour and the investigated organs.

### Study workflow

The published PBPK model [19] was implemented in the NONMEM software (version 7.5.1; ICON Development Solutions, Ellicott City, MD). The important sampling (IMP) method was used as the estimation method to fit the PBPK model parameters to the biokinetic data. The method was chosen because it is well-suited for complex models and produces reliable results for large and sparse data sets [27].

**Fig. 2:**
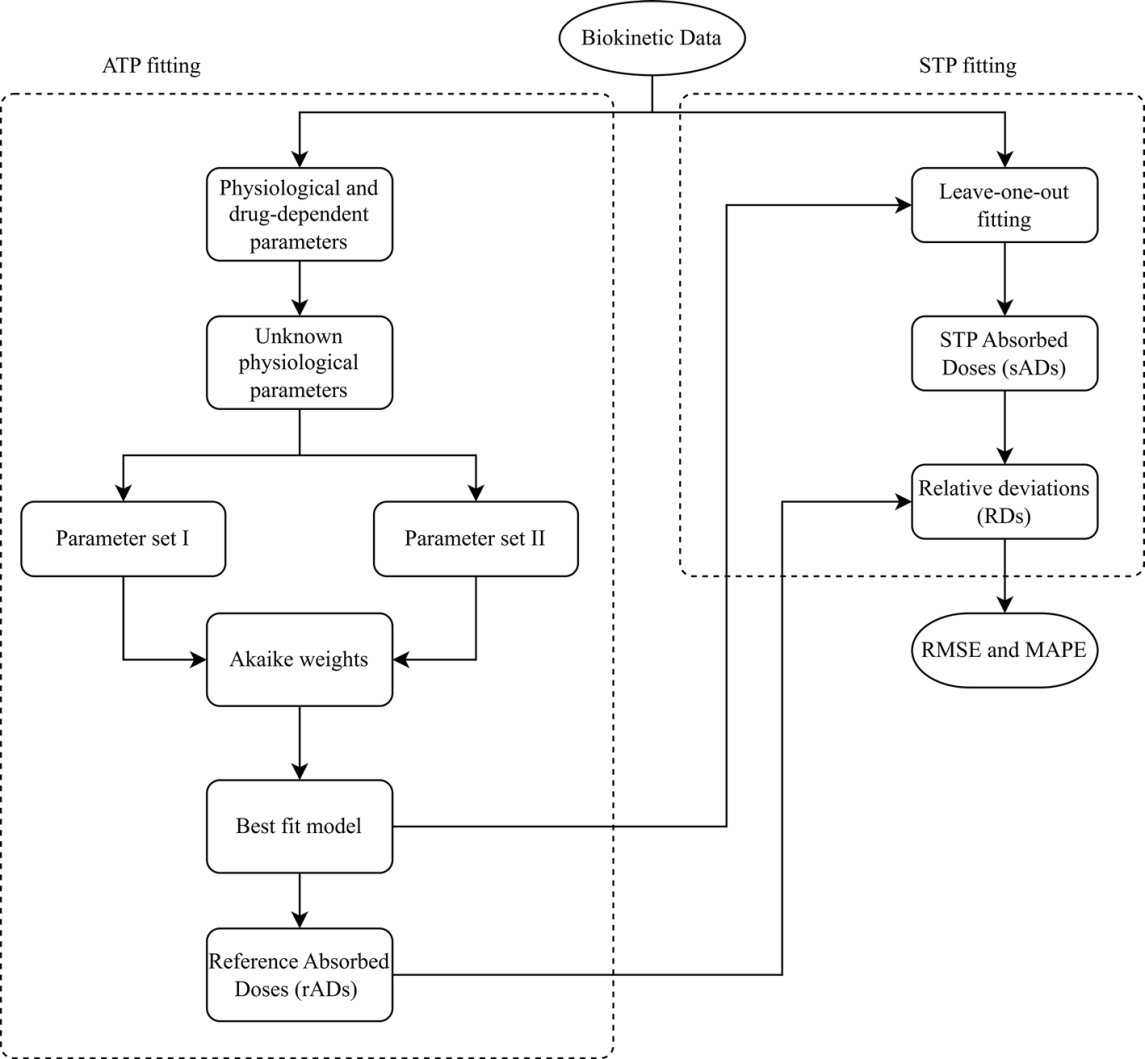
Workflow of the study. The parameter set of the PBPK model was adapted from Hardiansyah et al. [10] for setting I and fitted all the unknown parameter with the addition of the prior knowledge of blood flow rate to the tumour from the literature [19, 28, 29] for setting II, respectively. The best parameter set was selected based on the Akaike weights. The ADs obtained from best parameter set in ATP fitting were selected as the rADs. For STP dosimetry, the leave-one-out method was applied. The accuracy of STP dosimetry was evaluated through the RMSE and MAPE, which were calculated based on Eqs. (6) and (8)

Figure 2 shows the workflow of this study. The PBPK model consists of physiological and drug-dependent parameters. The physiological parameters easily measured were fixed to the individual values. Other parameters were taken from the literature (e.g., blood flow). The drug-dependent parameters that were reported in the literature (e.g., dissociation constant) were fixed to the reported value. Two sets of the fitted parameters were analysed: (setting I) the set of the fitted parameters used in our previous study [10] and (setting II) the set of the fitted parameters wherein all unknown parameters were fitted to the biokinetic data. This set was then supplemented with additional prior knowledge obtained from the literature [19, 28, 29]. For setting II, in our preliminary study, all unknown PBPK model parameters were fitted to the biokinetic data. However, the fitting did not pass the goodness of fit (GoF) test (data not shown). Therefore, prior knowledge of the tumour blood flow rate of 0.9 mL g^−1^ min^−1^ for meningioma [28] and 1.0 mL g^−1^ min^−1^ for NETs [29] were used. The prior knowledge was used as the tumour blood blow rate was difficult to estimate in some patients due to the earliest biokinetic measurement in our population being 2 h after injection [19]. The GoF criteria in all-time-point (ATP) fitting was an adequate visual inspection of the fitted curves, the relative standard errors (RSEs) of the FE and RE (inter- and intra-individual variabilities) of the PBPK model parameters < 50%, and the absolute value of the off-diagonal elements of the correlation matrix < 0.8 [19]. The best parameter setting was selected based on the Akaike weight values. The obtained ADs from ATP fitting were used as the reference (rADs).

For STP dosimetry, a leave-one-out Jackknife method was employed. In this method, the data of the investigated patients were fitted using STP data for each organ and excluding all data for blood serum as performed in the literature [10]. The absorbed doses obtained in STP dosimetry were designated as STP ADs (sADs).

The TIAC was calculated numerically from time zero to 30,000 min [10]. The values on individual TIACs’ standard errors were obtained using the error propagation method [14, 17] as follows:

3 $$\begin{array}{*{20}c} {\Delta {\text{TIAC}} = \sqrt {\mathop \sum \limits_{i} \left( {\Delta A\left( {t_{i} } \right)} \right)^{2} } } \\ \end{array}$$

where ΔTIACs are the uncertainties for the TIACs; and ${\Delta }A\left( {t_{i} } \right)$ represents the standard error of the activity at time point $t_{i}$, derived from the PBPK model prediction using the NONMEM software. The equations for the uncertainties of the RDs, RMSE, and MAPE are provided in the supplemental file part A: derivation of the uncertainties.

The self-absorbed dose was calculated for kidney and tumour. Self-absorbed dose factor for kidney was found from the literature [10, 19, 30] as 2.93 × 10^–5^ (Gy min^−1^ MBq^−1^) and 3.18 × 10^–5^ (Gy min^−1^.MBq^−1^) for male and female, respectively. The S value for tumours were interpolated based on the spherical tumour model provided in the OLINDA/EXM software [30], the S value for [^90^Y]Y-DOTA-TATE obtained based on Eq. (4) as described in the literature [10, 19, 31]

4 $$\begin{array}{*{20}c} {S_{TU \leftarrow TU} = \left( {\frac{0.28}{V} - \frac{{1.67 \times 10^{ - 3} }}{{V^{\frac{2}{3}} }} - \frac{0.82}{{V^{\frac{1}{3}} }}} \right) Gy\,\,{\text{min}}^{ - 1} \,\,{\text{MBq}}^{ - 1} } \\ \end{array}$$

with $V$ being the tumour volume in mL.

### Data analysis

The accuracy of the STP dosimetry was evaluated by calculating the relative deviations (RDs) of the sADs relative to the rADs using the following equation:

5 $$\begin{array}{*{20}c} {{\text{RD}} = \frac{{{\text{sADs}} - {\text{rADs}}}}{{{\text{rADs}}}}} \\ \end{array}$$

In this study, an acceptable estimation of sADs was defined as an absolute RD ≤ 10%. Note that the same RDs apply to TIACs and absorbed doses when assuming that the same S values are used in the ATP and STP methods. To determine the best time point to perform STP fitting, the root-mean-square error (RMSE) was calculated for each time point and organ according to [15, 32]:

6 $$\begin{array}{*{20}c} { {\text{RMSE}}_{j} = \sqrt {\left( {{\text{SD of RD}}_{j} } \right)^{2} + \left( {{\text{Mean of RD}}_{j} } \right)^{2} } } \\ \end{array}$$

with SD of ${\text{RD}}_{j}$ being the standard deviation of the RD at time point $j$ and mean of ${\text{RD}}_{j}$ being the average of the RD at time point $j$. Since the kidneys and tumours are the main of interest in therapeutical dosimetry, an RMSE_joint_ value which describes the combined RMSE from kidneys and tumours was calculated [10, 32]

7 $$\begin{array}{*{20}c} {{\text{RMSE}}_{{{\text{joint}}}} = \omega \cdot {\text{RMSE}}_{{{\text{kidneys}}}} + \left( {1 - \omega } \right) \cdot {\text{RMSE}}_{{{\text{tumour}}}} } \\ \end{array}$$

The weighting factor $\omega$ was set to 0/6, 1/6, 2/6, 3/6, 4/6, 5/6, and 6/6 to evaluate the sensitivity of ${\text{RMSE}}_{{{\text{joint}}}}$ to the choice of the $\omega$ value, as recommended in the literature [10]. The optimal time point for STP fitting was selected based on the smallest RMSE and ${\text{RMSE}}_{{{\text{joint}}}}$ value.

The mean absolute percentage error (MAPE) was also calculated to assess the accuracy of the STP dosimetry and to validate the RMSE value, as RMSE is more sensitive to outliers. MAPE was calculated as follows:

8 $$\begin{array}{*{20}c} {MAPE = \frac{1}{n}\mathop \sum \limits_{i = 1}^{n} \left| {\frac{{{\text{sAD}}_{i} - {\text{rAD}}_{i} }}{{{\text{rAD}}_{i} }}} \right| } \\ \end{array}$$

where $n$ is the number of the investigated patients in the study.

The precision of ADs was defined as the RSE, calculated as the ratio between the standard errors of ADs and the value of ADs.

## Results

The PBPK model with setting II is selected as the best set of the fitted parameters with the Akaike weight of nearly 100%. The ATP fitting with NLMEM yielded the precise estimation of the PBPK parameters, as indicated by the maximum RSE value of FE and RE of 35%. The values of FE and RE (including the RSEs) are presented in Table 1. In addition, the intra-individual variabilities of blood serum, whole body, kidney, spleen, liver, and tumour were 23%, 8.8%, 5.3%, 6.7%, 10% and 6.0%, respectively. The maximum absolute value of the off-diagonal elements in the correlation matrix for this model is 0.71. Therefore, the ATP fit passed the GoF test criteria. The biokinetic curves of the tumour and investigated organs from ATP fitting are presented in Fig. S1 of the supplemental file.

**Table 1:** The value of the FE, RE, intra-individual variability, and their corresponding RSEs from ATP fitting

Parameters	FE (%RSE)	RE (%RSE)
$\left[ {R_{{\text{K,0}}} } \right]\left( {\text{nmol/L}} \right)$	4.55 (15)	45 (5)
$\left[ {R_{{\text{L,0}}} } \right] \left( {\text{nmol/L}} \right)$	0.94 (15)	43 (8)
$\left[ {R_{{\text{S,0}}} } \right] \left( {\text{nmol/L}} \right)$	7.37 (19)	52 (5)
$\left[ {R_{{\text{TU,0}}} } \right] \left( {\text{nmol/L}} \right)$	15.9 (30)	101 (10)
$\left[ {R_{{\text{Rest,0}}} } \right] \left( {\text{nmol/L}} \right)$	0.36 (30)	97 (7)
$k_{{\text{on, Alb}}} \left( {\text{nmol/L}} \right)$	$5.50 \times 10^{ - 4}$ (18)	52 (16)
$\lambda_{{{\text{int}},{\text{ K}}}} \left( {{\text{min}}^{ - 1} } \right)$	$2.36 \times 10^{ - 3}$ (11)	28 (25)
$\lambda_{{{\text{int}},{\text{ TU}}}} \left( {{\text{min}}^{ - 1} } \right)$	$1.39 \times 10^{ - 3}$ (14)	37 (15)
$\lambda_{{{\text{deg}},{\text{ NT}}}} \left( {{\text{min}}^{ - 1} } \right)$	$1.01 \times 10^{ - 4}$ (10)	19 (35)
$\lambda_{{{\text{deg}},{\text{ TU}}}} \left( {{\text{min}}^{ - 1} } \right)$	$1.15 \times 10^{ - 4}$ (24)	36 (6)
*Intra-individual variability (the value of parameter* $a$ *in Eq. *2*)*		
Blood serum	23 (8)	
Whole body	8.8 (14)	
Kidney	5.3 (11)	
Spleen	6.7 (14)	
Liver	10 (9)	
Tumour	6.0 (10)	

The value inside the parenthesis is the RSEs for each fitted parameter. RE is given in percent of the coefficient of variation that describes the variation of the parameters’ value around the FE. Intra-individual variability is given as percent fractional standard deviation (FSD)

**Fig. 3:**
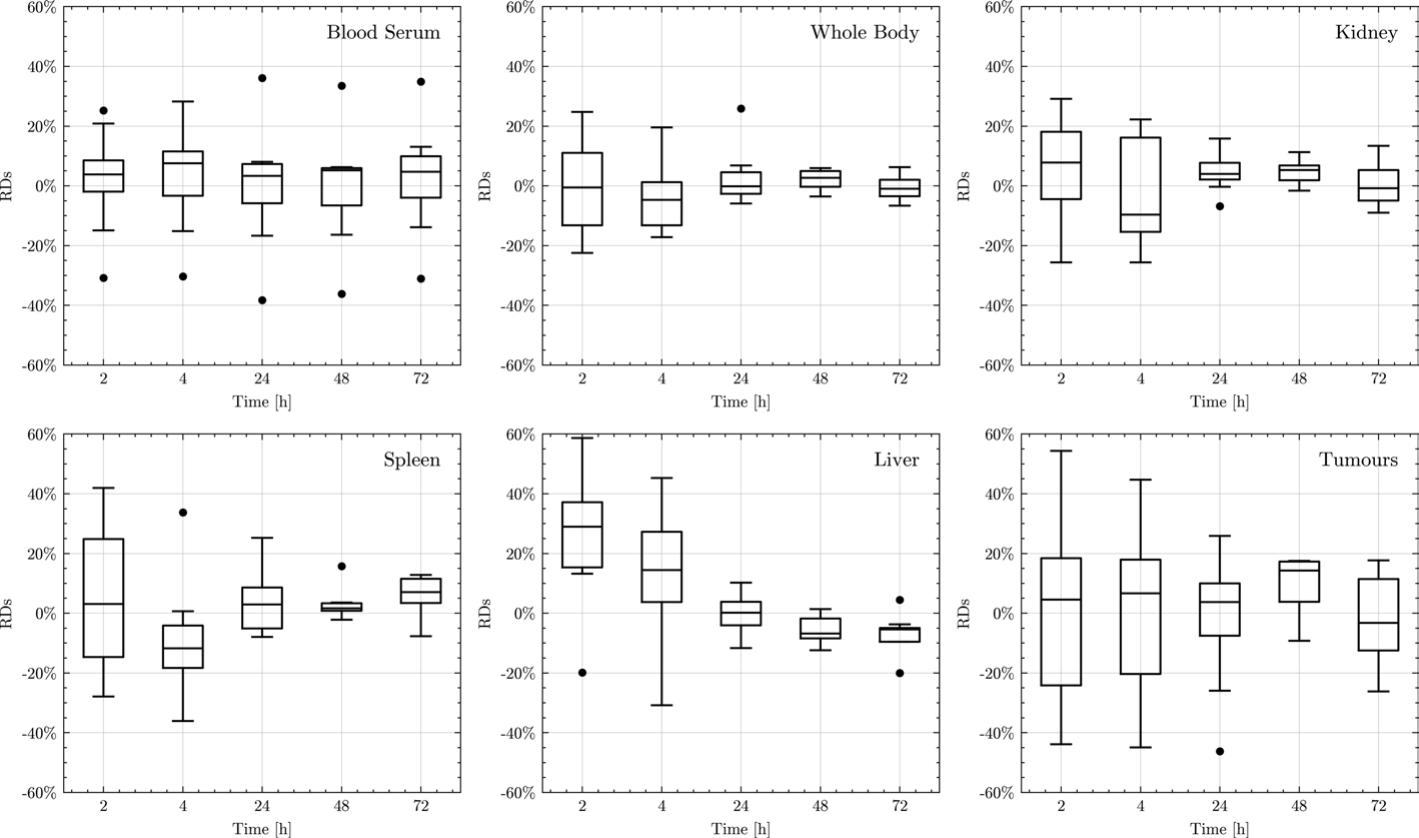
RDs of TIACs in STP compared to ATP fitting. Blood serum data of the specific STP patient were excluded in STP fitting. The RD for tumours of 80% and 233% (patient 4 at 4 h and 2 h after injection, respectively, tumour volume 2.8 mL) are excluded from the graph due to scale limit

Figure 3 presents the accuracy of STP NLMEM in calculating the TIACs for both tumour and investigated organs. Based on the median and range of RD values, time point 48 h after injection is the time point that yielded the lowest median and range values for kidneys and tumours. The median[min, max] of RD for kidneys and tumours were 5[− 2, 11]% and 14[− 9, 18]%, respectively.

Based on the RMSE value, time point 48 h after injection had the lowest RMSE for kidneys and tumours of (7 ± 2)% and (14 ± 4)%, respectively. For RMSE_joint_, it is shown that 48 h after injection consistently resulted in the lowest RMSE_joint_ for all $\omega$ values, as seen in Figure S3 in the supplemental file. Therefore, it can be inferred that time point 48 h after injection is the optimal time point to perform STP dosimetry using the PBPK model in this study. The MAPE evaluation also yielded 48 h after injection as the best time point, with the MAPE values for kidneys and tumours being (5 ± 2)% and (13 ± 4)%, respectively. The MAPE values of the other time points and organs are presented in Table S5 of the supplemental file.

**Fig. 4:**
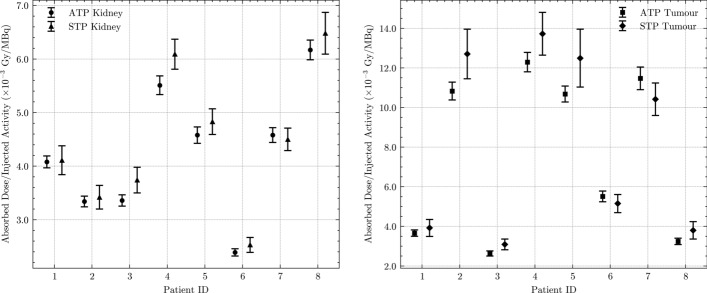
Individual ADs per injected activity for kidneys and tumours from ATP and STP dosimetry at 48 h after injection. The individual TIACs, obtained from the PBPK model fitting, were employed to calculate the individual value of ADs based on the MIRD formalism. The error bars represent the uncertainties of ADs calculated based on error propagation [14]

The accuracy and precision of the rADs and sADs are presented in Fig. 4. The sADs showed lower precision values than the rADs in all patients. For instance, the precision of the rADs and sADs at 48 h after injection for kidneys in terms of median[min, max] RSEs were 3[3, 3]% and 6[5, 6]%, respectively. Similar results were found for the tumours where the precision of the rADs and sADs at 48 h after injection were 4[4, 5]% and 9[8, 12]%, respectively.

## Discussion

Recently, we presented a method that employed a PBPK model, STP data, and NLMEM to enhance the accuracy of sADs [10]. The PBPK model with setting II resulted in a better fit than the PBPK model with setting I, as demonstrated by the Akaike weight value of nearly 100%. Precise estimation [23] of both the FE and RE in the PBPK model with setting II is shown in Table 1. In the previous study [10], the blood flow rate to the tumours was fitted. In this study, the blood flow rate to the tumours was fixed to the values reported in the literature [19, 28, 29]. The tissue uptake of DOTA-TATE is limited by blood flow. Therefore, blood flow to the tumour was identified as an important parameter for describing the pharmacokinetics of DOTA-TATE. However, the earliest imaging measurement was conducted at 2 h after injection in several patients, which made it difficult to estimate the blood flow to the tumour in the PBPK model. The kidneys and tumours internalisation rates, which were previously set to the population average values in the prior studies [10, 19], were fitted to account for potential individual variations. Our fitting result (Table 1) shows that the average ratio of kidneys’ internalisation rate $\left( {\lambda_{{{\text{int}},{\text{ K}}}} } \right)$ and tumours’ internalisation rate $\left( {\lambda_{{{\text{int}},{\text{ TU}}}} } \right)$ was 1.7, consistent with the literature [33]. The rest of the fitted parameter values were like those reported in the literature [10, 19].

Many possible sets of fitted parameters could be tested in the PBPK model. One approach that could be used to assess the influence of key parameters on the calculation of TIAC is global sensitivity analysis, as described in our previous publication, i.e., Hardiansyah et al. [34]. However, this study aimed to present the importance of the set of fitted parameters in the PBPK model. Therefore, a comprehensive screening of the effects of the set of fitted parameters on the model performance was not conducted in this study; comprehensive screening of various sets of fitted parameters was beyond the primary scope of this study.

Bonate et al. [24] suggested using different values of intra-individual variability for different population data, such as for the healthy volunteers and the analysed subjects, to account for the differences in kinetics. This variation in kinetics was also observed in different tumours and organs for a single patient, as shown in the literature [35], highlighting the different intra-individual variability for tumours and organs. In this study, we found that the RE intra-individual variabilities of blood serum, whole body, kidney, spleen, liver, and tumour for ATP fitting were 23%, 8.8%, 5.3%, 6.7%, 10% and 6.0%, respectively (Table 1). The results also show more accurate values (except for blood serum) than the previously published study [10], which only employed one intra-individual error model for all tumours and investigated organs and obtained an intra-individual variability of 20%. These findings support that modelling with more parameters, e.g. intra-individual variability for each organ, may lead to a better NLMEM performance as shown in our previous study [36].

The used PBPK model has shown good performance in describing the ATP biokinetic data (section Biokinetic Data) based on the GoF test (Table 1 and Fig. S1 supplement file). In STP fitting, the median RDs and RMSE_joint_ (Supplemental file Table S4 and Fig. S3) showed an acceptable estimation of sADs 48 h after injection. Identifying 48 h after injection as the optimal time point for STP dosimetry is in line with our previous study [10, 37], which had a comparable RD and RMSE. The RD for kidneys and tumours at 48 h after injection in this study were 5[− 2, 11]% and 14[− 9, 18]%, respectively, whereas in the prior study [10] were 5[1, 21]% and 2[− 15, 21]%, respectively. The RMSE between this study and Hardiansyah et al. [10] are similar and the values fall within the uncertainty range. There are differences between the individual RDs in this study (Fig. 3) and those reported in the previous study [10] due to the different sets of fitted parameters (Supplement file. Table S2). Nevertheless, the RMSEs in the population for kidneys and tumours were found to be similar to the previous study [10], i.e. the RMSE values fall within the 95% confidence interval; at 48 h after injection, this study: RMSE kidneys = (7 ± 2)%, RMSE tumours = (14 ± 4)% vs previous study [10]: RMSE kidney = 11%, RMSE tumours = 12%. Further investigation regarding the effect of the sets of fitted parameters on individual and population levels may be required in a large dataset to validate the findings.

The MAPE values confirm the RMSE, i.e. time point 48 h after injection is the optimal time point for STP dosimetry. The MAPE values at 48 h after injection for kidney and tumours are (5 ± 2)% and (13 ± 4)%, respectively. The Wilcoxon paired test shows no statistically significant difference in MAPE for STP dosimetry between 24 h, 48 h, and 72 h after injection for kidneys and between 48 and 72 h after injection for tumour, respectively. These results suggest that MAPE values are similar when performing STP dosimetry 24 h, 48 h, or 72 h after injection using the PBPK model in this study, although there may be a difference in individual RDs. However, this result, which shows no statistically significant difference, must be validated in a larger dataset.

According to several studies, one SPECT/CT measurement around 0.75 times the effective half-life (T_eff_) to 2.5 times T_eff_ can estimate sADs supposing mono-exponential biokinetics [38, 39]. For instance, Hou et al. demonstrated that STP dosimetry at 72 h after injection (~ 1.5T_eff_), supposing mono-exponential biokinetics in the kidney, resulted in sADs RD less than 10% for therapy with [^177^Lu]Lu-DOTA-TATE [40]. Similarly, Rinscheid et al. found that STP dosimetry at 52 h after injection (~ 1T_eff_ for tumours and ~ 1.3T_eff_ for kidneys) led to an acceptable estimation of sADs calculations with a RD of (− 2.8 ± 6.4)% for kidney and (− 14 ± 7.6)% for tumour [7]. By supposing the mono-exponential biokinetic, the biological half-life of DOTA-TATE in the kidneys was estimated to be 81 h [41] and the physical half-life of ^90^Y to be 64.2 h [19]. Therefore, the effective half-life of [^90^Y]Y-DOTA-TATE in kidneys was estimated to be 36 h. Thus, the optimal time point for sAD estimation, identified as 48 h after injection is around 1.3T_eff_, consistent with reported literature values [38, 39]. Despite the potential bias of the calculated RDs introduced by the mono-exponential fit function in rAD calculations, the optimal time points for conducting STP dosimetry using mono-exponential function [38] is similar to the optimal time point when using population-based model selection with the NLMEM result [15]. Consequently, our findings suggest that an acceptable estimation of ADs for [^90^Y]Y-DOTA-TATE in kidneys and tumours can be achieved using a PBPK model, the NLMEM, and STP biokinetic data from [^111^In]In-DOTA-TATE biokinetic measurements at 48 h after injection. The earliest measurement time point with relatively low MAPE values for the STP dosimetry in kidneys and tumours were 24 h and 48 h after injection, respectively (Tabel S5, supplement file). These results show that the STP dosimetry for tumours could be done at a later time point than the kidneys, which is in line with the previous finding in the literature [38].

The precision of the AD given as RSEs in kidneys and tumours was relatively high, as indicated by the maximum RSE of 5.1% (Fig. 4). As expected, there was a decrease in the precision for sAD in in comparison to rAD. The maximum RSE of sAD for kidneys and tumours at 48 h after injection was ~ 2.3 times greater than the maximum RSE of rAD. This is consistent with the findings that using fewer data points in fitting lowers the TIAC precision, and subsequently the ADs [16, 17]. Despite employing a different radiopharmaceutical and fitting method, Jundi et al. [16] also observed a similar pattern of decreased precision in STP TIAC RSEs compared to ATP TIAC RSEs for kidneys. The reported kidneys’ precision from ATP TIAC ranges from 0.8 to 49%, whereas the precision of STP TIAC ranges from 22 to 78%.

Precision information can be applied to determine the reliability of the AD estimation in clinical routine. Using the estimated ADs from ATP biokinetic data and STP data in multi-cycle therapy could be used as suggested in the literature [11–13]. Determining whether the ADs remain constant, decrease, or increase between cycles cannot be performed without knowing the uncertainties of individual ADs obtained from both ATP dosimetry and STP dosimetry in different cycles. The incorporation of uncertainty data, i.e., the standard error of the individual ADs, facilitates the application of several strategies to optimise the dosimetry protocols in instances when substantial disparities, or their absence, are present in ADs across treatment cycles. By having the uncertainty information of individual ADs, the physician can determine whether the ADs in multi-cycle therapy still similar or has been changed, this information can be used to adjust the injected activity in the subsequent cycle.

## Conclusions

In this study, we have presented a general method to calculate the RDs for accuracy analyses and uncertainty of individual ADs for precision analyses. The calculations were performed for ATP and STP fitting utilising a PBPK model within the NLMEM. The findings indicate a lower sAD precision in comparison to rAD. The uncertainties information of the individual ADs could be used to assess the reliability of the ADs estimation, which might be used to develop optimal strategies such as the determination of the administered activity in different therapy cycles within a combination of ATP and STP dosimetry protocols.

## Supplementary Information

Additional file 1.

## Data Availability

The used data are available from the corresponding author upon reasonable request.

## Abbreviations

ATP: All-time-point
FE: Fixed-effects
MAPE: Mean absolute percentage error
MRT: Molecular radiotherapy
NLMEM: Non-linear mixed-effects modelling
PBPK: Physiologically-based pharmacokinetic
RD: Relative deviations
RE: Random-effects
RMSE: Root-mean-square error
RSE: Relative standard error
STP: Single-time-point
TIAC: Time-integrated activity coefficient

## References

[1] Glatting G, Bardiès M, Lassmann M. Treatment planning in molecular radiotherapy. Z Med Phys. 2013;23:262–9. 10.1016/J.Zemedi.2013.03.005. 23597414 10.1016/j.zemedi.2013.03.005

[2] Hardiansyah D, Maass C, Attarwala A, Müller B, Kletting P, Mottaghy F, et al. The role of patient-based treatment planning in peptide receptor radionuclide therapy. Eur J Nuclear Med Mol Imaging. 2016;43:871–80. 10.1007/S00259-015-3248-6. 10.1007/s00259-015-3248-6 26577941

[3] Lassmann M, Chiesa C, Flux G, Bardies M, Committee E. EANM dosimetry committee guidance document: good practice of clinical dosimetry reporting. Eur J Nucl Med Mol Imaging. 2011;38:192–200. 10.1007/S00259-010-1549-3. 20799035 10.1007/s00259-010-1549-3

[4] Vergnaud L, Dewaraja Y, Giraudet A-L, Badel J-N, Sarrut D. A review of ^177^Lu dosimetry workflows: how to reduce the imaging workloads? EJNMMI Phys. 2024;11:65. 10.1186/S40658-024-00658-8. 39023648 10.1186/s40658-024-00658-8 PMC11554969

[5] Spink S, Gillett D, Heard S, Harper I, Casey R, Aloj L. Estimation of kidney doses from [^177^lu]Lu-DOTA-TATE PRRT using single time point post-treatment SPECT/CT. Ejnmmi Phys. 2024. 10.1186/S40658-024-00665-9. 39052172 10.1186/s40658-024-00665-9 PMC11272758

[6] Peterson AB, Mirandi DM, Dewaraja YK. Accuracy and uncertainty analysis of reduced time point imaging effect on time-integrated activity for ^177^Lu-DOTATATE PRRT In patients and clinically realistic simulations. EJNMMI Res. 2023. 10.1186/S13550-023-01007-Z. 37306783 10.1186/s13550-023-01007-z PMC10260735

[7] Rinscheid A, Kletting P, Eiber M, Beer A, Glatting G. Influence of sampling schedules on [^177^Lu]Lu-PSMA dosimetry. EJNMMI Phys. 2020. 10.1186/S40658-020-00311-0. 32556844 10.1186/s40658-020-00311-0 PMC7300169

[8] Siebinga H, De Wit-Van-De-Veen B, Stokkel M, Huitema A, Hendrikx J. Current use and future potential of (physiologically based) pharmacokinetic modelling of radiopharmaceuticals: a review. Theranostics. 2022;12:7804–20. 10.7150/Thno.77279. 36451855 10.7150/thno.77279 PMC9706588

[9] Deepika D, Kumar V. The role of “physiologically based pharmacokinetic model (PBPK)” new approach methodology (Nam). Pharmac Environ Chem Risk Assess. 2023. 10.3390/Ijerph20043473. 10.3390/ijerph20043473 PMC9966583 36834167

[10] Hardiansyah D, Riana A, Beer A, Glatting G. Single-time-point estimation of absorbed doses In PRRT Using A Non-linear mixed-effects model. Z Med Phys. 2023;33:70–81. 10.1016/j.zemedi.2022.06.004 35961809 10.1016/j.zemedi.2022.06.004 PMC10082376

[11] Willowson KP, Eslick E, Ryu H, Poon A, Bernard EJ, Bailey DL. Feasibility and accuracy of single time point imaging for renal dosimetry following ^177^Lu-DOTATATE (‘lutate’) therapy. EJNMMI Phys. 2018. 10.1186/s40658-018-0232-9. 30569328 10.1186/s40658-018-0232-9 PMC6300448

[12] Peters S, Hofferber R, Privé B, De Bakker M, Gotthardt M, Janssen M, et al. [^68^Ga]Ga-PSMA-11 PET imaging as a predictor for absorbed doses in organs at risk and small lesions in [^177^Lu]Lu-PSMA-617 treatment. Eur J Nuclear Med Mol Imaging. 2022;49:1101–12. 10.1007/S00259-021-05538-2. 10.1007/s00259-021-05538-2 PMC8921092 34623453

[13] Brosch-Lenz J, Delker A, Völter F, Unterrainer L, Kaiser L, Bartenstein P, et al. toward single-time-point image-based dosimetry of ^177^Lu-PSMA-617 therapy. J Nuclear Med. 2023;64:767–74. 10.2967/Jnumed.122.264594. 10.2967/jnumed.122.264594 PMC10152120 36657980

[14] Gear J, Cox M, Gustafsson J, Gleisner K, Murray I, Glatting G, et al. EANM practical guidance on uncertainty analysis for molecular radiotherapy absorbed dose calculations. Eur J Nuclear Med Mol Imaging. 2018;45:2456–74. 10.1007/S00259-018-4136-7. 10.1007/s00259-018-4136-7 PMC6208822 30218316

[15] Hardiansyah D, Yousefzadeh-Nowshahr E, Kind F, Beer A, Ruf J, Glatting G, et al. Single-time-point renal dosimetry using nonlinear mixed-effects modeling and population-based model selection In [^177^Lu]Lu-PSMA-617 therapy. J Nucl Med. 2024;65:566–72. 10.2967/Jnumed.123.266268. 38423787 10.2967/jnumed.123.266268

[16] Jundi A, Naqiyyun M, Patrianesha B, Mu’minah I, Riana A, Hardiansyah D. Uncertainty analysis of time-integrated activity coefficient in single-time-point dosimetry using Bayesian fitting method. Nuclear Med Mol Imaging. 2024;58:120–8. 10.1007/S13139-024-00851-8. 10.1007/s13139-024-00851-8 PMC11018592 38633290

[17] Peters S, Mink M, Privé B, De-Bakker M, De Lange F, Muselaers C, et al. Optimization of the radiation dosimetry protocol in lutetium-177-psma therapy: toward clinical implementation. Ejnmmi Res. 2023. 10.1186/S13550-023-00952-Z. 36692682 10.1186/s13550-023-00952-z PMC9873880

[18] Begum N, Thieme A, Eberhardt N, Tauber R, D’alessandria C, Beer A, et al. The effect of total tumor volume on the biologically effective dose to tumor and kidneys for ^177^Lu-labeled psma peptides. J Nuclear Med. 2018;59:929–33. 10.2967/Jnumed.117.203505. 10.2967/jnumed.117.203505 29419479

[19] Kletting P, Kull T, Maa C, Malik N, Luster M, Beer A, et al. Optimized peptide amount and activity for ^90^Y-labeled dotatate therapy. J Nuclear Med. 2016;57:503–8. 10.2967/Jnumed.115.164699. 10.2967/jnumed.115.164699 26678617

[20] Geworski L, Schaefer A, Knoop B, Pinkert J, Plotkin M, Kirsch C. Physical aspects of scintigraphy-based dosimetry for nuclear medicine therapy. Nuklearmedizin. 2010;49:85–95. 10.3413/Nukmed-0283. 20505893 10.3413/nukmed-0283

[21] Siegel J, Thomas S, Stubbs J, Stabin M, Hays M, Koral K, et al. Mird pamphlet No. 16: techniques for quantitative radiopharmaceutical biodistribution data acquisition and analysis for use in human radiation dose estimates. J Nucl Med. 1999;40:37s–61s. 10025848

[22] Hardiansyah D, Riana A, Beer A, Glatting G. Single-time-point dosimetry using model selection and nonlinear mixed-effects modelling: a proof of concept. Ejnmmi Phys. 2023;10:12. 10.1186/S40658-023-00530-1. 36759362 10.1186/s40658-023-00530-1 PMC9911583

[23] Owen J, Fiedler-Kelly J. Introduction to population pharmacokinetic/pharmacodynamic analysis with nonlinear mixed effects models. Wiley; 2014. 10.1038/psp.2014.51 PMC5684957 25531560

[24] Bonate PI. Nonlinear mixed effects models: theory. US: springer; 2011. p. 233–301.

[25] Bauer R. Nonmem tutorial part I: description of commands and options, with simple examples of population analysis. Cpt Pharmacometr Syst Pharmacol. 2019;8:525–37. 10.1002/Psp4.12404. 10.1002/psp4.12404 PMC6709426 31056834

[26] Rinscheid A, Kletting P, Eiber M, Beer A, Glatting G. Technical note: optimal sampling schedules for kidney dosimetry based on the hybrid planar/spect method in ^177^Lu-PSMA therapy. Med Phys. 2019;46:5861–6. 10.1002/Mp.13846. 31587333 10.1002/mp.13846

[27] Bauer R. Nonmem tutorial part ii: estimation methods and advanced examples. Cpt Pharmacometr Syst Pharmacol. 2019;8:538–56. 10.1002/Psp4.12422. 10.1002/psp4.12422 PMC6709422 31044558

[28] Kimura H, Takeuchi H, Koshimoto Y, Arishima H, Uematsu H, Kawamura Y, et al. Perfusion imaging of meningioma by using continuous arterial spin-labeling: comparison with dynamic susceptibility-weighted contrast-enhanced MR images and histopathologic features. Ajnr Am J Neuroradiol. 2006;27:85–93. 16418363 PMC7976105

[29] Guyennon A, Mihaila M, Palma J, Lombard-Bohas C, Chayvialle J, Pilleul F. Perfusion characterization of liver metastases from endocrine tumors: computed tomography perfusion. World J Radiol. 2010;2:449–54. 10.4329/Wjr.V2.I11.449. 21179313 10.4329/wjr.v2.i11.449 PMC3006484

[30] Stabin M, Sparks R, Crowe E. Olinda/Exm: the second-generation personal computer software for internal dose assessment in nuclear medicine. J Nucl Med. 2005;46:1023–7. 15937315

[31] Mg S, Ja S. Physical models and dose factors for use in internal dose assessment. Health Phys. 2003;85:294–310. 10.1097/00004032-200309000-00006. 12938720 10.1097/00004032-200309000-00006

[32] Rinscheid A, Lee J, Kletting P, Beer A, Glatting G. A simulation-based method to determine optimal sampling schedules for dosimetry in radioligand therapy. Z Med Phys. 2019;29:314–25. 10.1016/J.Zemedi.2018.12.001. 30611606 10.1016/j.zemedi.2018.12.001

[33] Antunes P, Ginj M, Zhang H, Waser B, Baum R, Reubi J, et al. Are radiogallium-labelled dota-conjugated somatostatin analogues superior to those labelled with other radiometals? Eur J Nucl Med Mol Imaging. 2007;34:982–93. 10.1007/S00259-006-0317-X. 17225119 10.1007/s00259-006-0317-x

[34] Hardiansyah D, Kletting P, Begum N, Eiber M, Beer A, Pawiro S, et al. Important pharmacokinetic parameters for individualization of ^177^Lu-PSMA therapy: a global sensitivity analysis for a physiologically-based pharmacokinetic model. Med Phys. 2021;48:556–68. 10.1002/Mp.14622. 33244792 10.1002/mp.14622

[35] Siebinga H, Privé B, Peters S, Nagarajah J, Dorlo T, Huitema A, et al. Population pharmacokinetic dosimetry model using imaging data to assess variability in pharmacokinetics of ^177^Lu-PSMA-617 in prostate cancer patients. Cpt Pharmacometr Syst Pharmacol. 2023;12:1060–71. 10.1002/Psp4.12914. 10.1002/psp4.12914 PMC10431047 36760133

[36] Hardiansyah D, Riana A, Kletting P, Zaid N, Eiber M, Pawiro S, et al. A population-based method to determine the time-integrated activity in molecular radiotherapy. EJNMMI Phys. 2021;8:82. 10.1186/S40658-021-00427-X. 34905131 10.1186/s40658-021-00427-x PMC8671591

[37] Hardiansyah D, Riana A, Beer A, Glatting G. Single-time-point dosimetry using model selection and nonlinear mixed-effects modelling: a proof of concept. Ejnmmi Phys. 2023. 10.1186/S40658-023-00530-1. 36759362 10.1186/s40658-023-00530-1 PMC9911583

[38] Hänscheid H, Lapa C, Buck A, Lassmann M, Werner R. Dose mapping after endoradiotherapy with ^177^Lu-DOTATATE/DOTATOC By A single measurement after 4 days. J Nucl Med. 2018;59:75–81. 10.2967/Jnumed.117.193706. 28588150 10.2967/jnumed.117.193706

[39] Madsen M, Menda Y, O’dorisio T, O’dorisio M. Technical note: single time point dose estimate for exponential clearance. Med Phys. 2018;45:2318–24. 10.1002/Mp.12886. 29577338 10.1002/mp.12886 PMC5948162

[40] Hou X, Brosch J, Uribe C, Desy A, Boning G, Beauregard J-M, et al. Feasibility of single-time-point dosimetry for radiopharmaceutical therapies. J Nuclear Med. 2020. 10.2967/Jnumed.120.254656. 10.2967/jnumed.120.254656 PMC8882881 33127625

[41] Guerriero F, Me F, Botta F, Fioroni F, Grassi E, Versari A, et al. Kidney dosimetry In ^177^Lu And ^90^Y peptide receptor radionuclide therapy: influence of image timing, time-activity integration method and risk factors. Biomed Res Int. 2013;2013:1–12. 10.1155/2013/935351. 10.1155/2013/935351 PMC3705840 23865075

