# Supplementary material for: Accuracy and precision analyses of single-time-point dosimetry utilising physiologically-based pharmacokinetic modelling and non-linear mixed-effects modelling
Source: EJNMMI Phys. 2025 Mar 26;12:26. doi: 10.1186/s40658-025-00726-7 (PMC11947405; doi:10.1186/s40658-025-00726-7)
Supplement: Supplementary file 1 — Additional file 1. [file 40658_2025_726_MOESM1_ESM.docx]

# **Supplemental File**

**Accuracy and Precision Analyses of Single-Time-Point Dosimetry Utilising Physiologically-Based Pharmacokinetic Modelling and Nonlinear Mixed-Effects Modelling**

Indra Budiansah^1$^, Deni Hardiansyah^1$*^, Ade Riana^1,2^, Supriyanto Ardjo Pawiro^1^, Ambros J. Beer^3^, Gerhard Glatting^3,4^

*^1^Medical Physics and Biophysics Division, Physics Department, Faculty of Mathematics and Natural Sciences, Universitas Indonesia, Depok, 16424, Indonesia*

*^2^Radiation Protection and compliance Testing Laboratory, Medical Devices and Facilities Safety Centre, Jakarta, Indonesia*

*^3^Department of Nuclear Medicine, Ulm University, Ulm, Germany.*

*^4^Medical Radiation Physics, Department of Nuclear Medicine, Ulm University, Ulm,* *Germany.*

^$^Equal contribution

*Corresponding Author:

Dr. sc. hum. Deni Hardiansyah

Medical Physics and Biophysics Division, Physics Department, Faculty of Mathematics and Natural Sciences, Universitas Indonesia, Depok, Indonesia

Email: [denihardiansyah@ui.ac.id](mailto:denihardiansyah@ui.ac.id)

**Part A: Derivation of the Uncertainties**

**Relative Deviations (RDs)**

Suppose the obtained sTIAC and rTIAC are $\left( a\pm\Delta a \right)$ and $\left( b\pm\Delta b \right)$, respectively. The RDs were calculated based on the Eq. (5). The uncertainty of RD can be obtained as follows:

| $\text{d RD}=\sqrt{\left( \frac{\partial\text{RD}}{\partial a}\text{d}a \right)^{2}+\left( \frac{\partial\text{RD}}{\partial b}\text{d}b \right)^{2}}$ $\Delta\text{RD}=\sqrt{\left( \frac{\text{d}a}{b} \right)^{2}+\left( \frac{-b\cdot\text{d}b-\left( a-b \right)\text{d}b}{b^{2}} \right)^{2}}$ $\Delta\text{RD}=\sqrt{\left( \frac{\text{d}a}{b} \right)^{2}+\left( \frac{-a\cdot\text{d}b}{b^{2}} \right)^{2}}$ $\Delta\text{RD}=\sqrt{\left( \frac{\Delta a}{b} \right)^{2}+\left( \frac{a\Delta b}{b^{2}} \right)^{2}}$ | (S1) |
| --- | --- |

**Mean and Standard Deviation uncertainties**

The mean RD (let’s assume it as $m$) and its’ uncertainties $\left( \Delta m \right)$ can be calculated as follows:

$$\begin{aligned} m=\sum_{i=1}^{n} \frac{\text{RD}_{i}}{n}\#\left( S2 \right) \end{aligned}$$

with $i$ being the patient ID and $n$ is the total number of the patients. The $\Delta m$ can be calculated as:

| $\Delta m=\sqrt{\sum_{i=1}^{n} \left( \frac{\partial m}{\partial\text{RD}_{i}}\text{d}\text{ RD}_{i} \right)^{2}}$ $\Delta m=\sqrt{\sum_{i=1}^{n} \left( \frac{\partial}{\partial\text{RD}_{i}}\left( \sum_{j=1}^{n} \frac{\text{RD}_{j}}{n} \right)\text{dRD}_{i} \right)^{2}}$ $\Delta m=\sqrt{\sum_{i=1}^{n} \left( \sum_{j=1}^{n} \frac{\delta_{ij} \text{dRD}_{j}}{n} \right)^{2}}$ $\Delta m=\sqrt{\sum_{i=1}^{n} \left( \frac{\Delta\text{RD}_{i}}{n} \right)^{2}}$ | (S3) |
| --- | --- |

The standard deviation (SD) of the sample calculated as

$$\begin{aligned} \text{SD}=\sqrt{\sum_{i=1}^{n} \frac{\left( \text{RD}_{i}-m \right)^{2}}{n-1}}\#\left( S4 \right) \end{aligned}$$

where $m$ is the mean of RD as defined in Eq. (S2). The uncertainty of SD can be calculated as:

$$\begin{aligned} \Delta SD=\sqrt{\sum_{i=1}^{n} \left( \frac{\partial SD_{i}}{\partial RD_{i}}\mathrm{dR}D_{i} \right)} \#\left( S5 \right) \end{aligned}$$

Let calculate the $\frac{\partial SD_{i}}{\partial RD_{I}}$ to simplify Eq. (S5)

$$\frac{\partial\text{SD}}{\partial\text{RD}_{i}}=\frac{\partial}{\partial\text{RD}_{i}}\sqrt{\sum_{j=1}^{n} \frac{\left( \text{RD}_{j}-m \right)^{2}}{n-1}}$$

$$\boldsymbol{=}\frac{1}{2\sqrt{\sum_{j=1}^{n} \frac{\left( \text{RD}_{j}-m \right)^{2}}{n-1}}}\frac{\partial}{\partial\text{RD}_{i}}\left[ \sum_{j=1}^{n} \frac{\left( \text{RD}_{j}-m \right)^{2}}{n-1} \right]$$

$$\boldsymbol{=}\frac{1}{2\cdot\text{SD}}\sum_{j=1}^{n} \frac{1}{n-1}\cdot2\left( \text{RD}_{j}-m \right)\cdot\frac{\partial}{\partial\text{RD}_{i}}\left( \text{RD}_{j}-m \right)$$

$$\boldsymbol{=}\frac{1}{2\cdot\text{SD}}\sum_{j=1}^{n} \frac{1}{n-1}\cdot2\left( \text{RD}_{j}-m \right)\cdot\left( \delta_{ij}-\frac{1}{n} \right)$$

$$\boldsymbol{=}\frac{1}{\left( n-1 \right)\cdot\text{SD}}\sum_{j=1}^{n} \left( \text{RD}_{j}-m \right)\left( \delta_{ij}-\frac{1}{n} \right)$$

$$\boldsymbol{=}\frac{1}{\left( n-1 \right)\cdot\text{SD}}\left( \left( \sum_{j=1}^{n} \text{RD}_{j}\delta_{ij} \right)-\left( \frac{1}{n}\sum_{j=1}^{n} \text{RD}_{j} \right)-m+\frac{1}{n}\sum_{j=1}^{n} m \right)$$

$$\boldsymbol{=}\frac{\text{RD}_{i}-m}{\left( n-1 \right)\cdot\text{SD}}$$

Therefore, the uncertainty for SD can be written as:

$$\begin{aligned} \Delta\text{SD}=\sqrt{\sum_{i=1}^{n} \left[ \frac{\text{RD}_{i}-m}{\left( n-1 \right)\cdot\text{SD}}\cdot\Delta\text{RD}_{i} \right]^{2}}\#\left( S6 \right) \end{aligned}$$

**Root-mean-square error (RMSE) uncertainty**The RSME was calculated based on Eq. (6). The uncertainty of the RMSE $\left( \Delta\text{RMSE} \right)$ was calculated as:

| $\Delta\text{RMSE}=\sqrt{\left( \frac{\partial\text{RMSE}}{\partial m} \text{d}m \right)^{2}+\left( \frac{\partial\text{RMSE}}{\partial\text{SD}} \text{dSD} \right)^{2}}$ $\Delta\text{RMSE}=\sqrt{\left( \frac{m}{\sqrt{m^{2}+\text{SD}^{2}}}\Delta m \right)^{2}+\left( \frac{\text{SD}}{\sqrt{m^{2}+\text{SD}^{2}}}\Delta\text{SD} \right)^{2}}$ $\Delta\text{RMSE}=\sqrt{\left( \frac{m}{\text{RMSE}}\Delta m \right)^{2}+\left( \frac{\text{SD}}{\text{RMSE}}\Delta\text{SD} \right)^{2}}$ | (S7) |
| --- | --- |

**Mean Absolute Percentage Error (MAPE) uncertainty**

The MAPE calculated utilising Eq. (8), its’ uncertainty was derived as follows:

$$\Delta\text{MAPE}=\sqrt{\sum_{i=1}^{n} \left( \frac{\partial\text{MAPE}}{\partial a_{i}} \text{d}a_{i} \right)^{2}+\sum_{i=1}^{n} \left( \frac{\partial\text{MAPE}}{\partial b_{i}} \text{d}b_{i} \right)^{2}}$$

let’s solve step-by-step

$$\frac{\partial\text{MAPE}}{\partial a_{i}}=\frac{\partial}{\partial a_{i}}\left\{ \frac{1}{n}\sum_{j=1}^{n} \frac{\left| a_{j}-b_{j} \right|}{b_{j}} \right\}$$

$$\frac{\partial\text{MAPE}}{\partial a_{i}}=\frac{\partial}{\partial a_{i}}\left\{ \frac{1}{n}\sum_{j=1}^{n} \frac{\sqrt{\left( a_{j}-b_{j} \right)^{2}}}{b_{j}} \right\}$$

$$\frac{\partial\text{MAPE}}{\partial a_{i}}=\frac{1}{n}\sum_{j=1}^{n} \frac{1}{2b_{j}\sqrt{\left( a_{j}-b_{j} \right)^{2}}}\cdot\frac{\partial}{\partial a_{i}}\left( a_{j}-b_{j} \right)^{2}$$

$$\frac{\partial\text{MAPE}}{\partial a_{i}}=\frac{1}{n}\sum_{j=1}^{n} \frac{2\left( a_{j}-b_{j} \right)}{2b_{j}\sqrt{\left( a_{j}-b_{j} \right)^{2}}}\cdot\delta_{ij}$$

$$\frac{\partial\text{MAPE}}{\partial a_{i}}=\frac{1}{n}\frac{a_{i}-b_{i}}{b_{i}\left| a_{i}-b_{i} \right|}$$

for parameter $b_{i}$ it can be derived as follows:

$$\frac{\partial\text{MAPE}}{\partial b_{i}}=\frac{\partial}{\partial b_{i}}\left\{ \frac{1}{n}\sum_{j=1}^{n} \frac{\left| a_{j}-b_{j} \right|}{b_{j}} \right\}$$

$$\frac{\partial\text{MAPE}}{\partial b_{i}}=\frac{1}{n}\sum_{j=1}^{n} \frac{\left\{ \frac{\partial}{\partial b_{i}}\left( \left| a_{j}-b_{j} \right| \right) \right\}b_{j}-\left| a_{j}-b_{j} \right|\delta_{ij}}{b_{j}^{2}}$$

$$\frac{\partial\text{MAPE}}{\partial b_{i}}=\frac{1}{n}\sum_{j=1}^{n} \frac{\frac{a_{j}-b_{j}}{\left| a_{j}-b_{j} \right|}b_{j}\left( -\delta_{ij} \right)-\left| a_{j}-b_{j} \right|\delta_{ij}}{b_{j}^{2}}$$

$$\frac{\partial\text{MAPE}}{\partial b_{i}}=\frac{1}{n}\left( -\frac{a_{i}-b_{i}}{b_{i}\left| a_{i}-b_{i} \right|}-\frac{\left| a_{i}-b_{i} \right|}{b_{i}^{2}} \right)$$

$$\frac{\partial\text{MAPE}}{\partial b_{i}}=\frac{1}{n}\left( \frac{-a_{i}b_{i}+b_{i}^{2}-a_{i}^{2}+2a_{i}b_{i}-b_{i}^{2}}{b_{i}^{2}\left| a_{i}-b_{i} \right|} \right)$$

$$\frac{\partial\text{MAPE}}{\partial b_{i}}=\frac{1}{n}\left( \frac{a_{i}\left( b_{i}-a_{i} \right)}{b_{i}^{2}\left| a_{i}-b_{i} \right|} \right)$$

Therefore, the uncertainty of MAPE can be written as follows:

| $\Delta\text{MAPE}=\sqrt{\sum_{i=1}^{n} \left( \frac{1}{n}\frac{a_{i}-b_{i}}{b_{i}\left\vert a_{i}-b_{i} \right\vert}\Delta a_{i} \right)^{2}+\sum_{i=1}^{n} \left( \frac{1}{n}\frac{a_{i}\left( b_{i}-a_{i} \right)}{b_{i}^{2}\left\vert a_{i}-b_{i} \right\vert}\Delta b_{i} \right)^{2}}$ $\Delta\text{MAPE}=\sqrt{\sum_{i=1}^{n} \left( \frac{\Delta a_{i}}{nb_{i}} \right)^{2}+\sum_{i=1}^{n} \left( \frac{a_{i}\Delta b_{i}}{nb_{i}^{2}} \right)^{2}}$ | (S8) |
| --- | --- |

**Part B: Supplemental Table**

Table S1. Parameter definition of the used physiologically-based pharmacokinetic (PBPK) model, adapted from the literature supplemental files [1]

| **Variable** | | **Value** | **Unit** |
| --- | --- | --- | --- |
| $k_{\mathrm{on}}$ | Association rate | ${k_{\mathrm{off}}}/{K_{D}}$ | $L\cdot nmol^{-1}\cdot\min^{-1}$ |
| $k_{\mathrm{off}}$ | Dissociation rate | 0.04 | $\min^{-1}$ |
| $k_{D}$ | Dissociation constant | 0.4 | $nmol\cdot L^{-1}$ |
| $\lambda_{\mathrm{phy}}$ | Physical decay of ${}^{111}\mathrm{In}$ and ${}^{90}Y$ | $1.71\times{10}^{-4}$ and $1.80\times{10}^{-4}$ | $\min^{-1}$ |
| $\mathrm{BW}$ | Body weight | Individually measured | kg |
| $H$ | Hematocrit | Individually measured | unity |
| $F$ | Flow total serum | $V_{p}\cdot\frac{1.23}{\min^{b}}$ | $L\cdot\min^{-1}$ |
| $V_{p}$ | Volume of total body serum |  | $L$ |
| **Tumour** | | | |
| $V_{TU,total}$ | Total volume of tumour | Measured | $L$ |
| $V_{TU,int}$ | Interstitial space of tumour | $v_{TU,int}\cdot V_{TU,total}$ | $L$ |
| $V_{TU,v}$ | Vascular space of tumour | $v_{TU,v}\cdot V_{TU,total}$ | $L$ |
| $v_{TU,int}$ | Interstitial space fraction of total tumour | - 1. for NET   0.23 for meningioma | unity |
| $v_{TU,v}$ | Vascular fraction of total tumour | 0.1 for NET  0.11 for meningioma | unity |
| $F_{\mathrm{TU}}$ | Serum flow tumour | $f_{\mathrm{TU}}\cdot\left( 1-H \right)\cdot V_{TU,total}$ | $L\cdot\min^{-1}$ |
| $f_{\mathrm{TU}}$ | Serum flow density tumour | 1.0 for NET  0.9 for meningioma | $\mathrm{mL}\cdot\min^{-1}\cdot g^{-1}$ |
| $PS_{\mathrm{TU}}$ | Permeability surface area product | $k_{\mathrm{TU}}\cdot V_{TU,total}$ | $\mathrm{mL}\cdot\min^{-1}$ |
| $k_{\mathrm{TU}}$ | Permeability surface area product per unit mass (scaled for molecule size of DOTATATE) | 0.2 for NET  0.31 for meningioma | $\mathrm{mL}\cdot\min^{-1}\cdot g^{-1}$ |
| $\left[ R_{\mathrm{TU},0} \right]$ | sst2 receptor density tumour | fitted | $nmol\cdot L^{-1}$ |
| $R_{\mathrm{TU},0}$ | sst 2 receptor number tumour | $\left[ R_{\mathrm{TU},0} \right]\cdot V_{TU,total}$ | $\mathrm{nmol}$ |
| $\lambda_{int,TU}$ | Internalization rate sst2 tumour | fitted | $\min^{-1}$ |
| $\lambda_{deg, TU}$ | Degradation rate of tumour | fitted | $\min^{-1}$ |
| **Measured Organ** | | | |
| $V_{L,total}$ | Volume total liver | Individually measured | $L$ |
| $V_{S,total}$ | Volume total spleen | Individually measured | $L$ |
| $V_{K,total}$ | Volume total kidney | Individually measured | $L$ |
| $V_{i,v}$ | Vascular (serum) volume organ liver, spleen, kidney | $V_{i,v}\cdot V_{i,total}$ | $L$ |
| $V_{i,int}$ | Interstitial volume liver, spleen, kidney | $V_{i,int}\cdot V_{i,total}$ | $L$ |
| $V_{K,intra}$ | Volume intercellular kidney | $\left( V_{K,total}-V_{K,int}-V_{K,v} \right)\cdot\frac{2}{3}$ | $L$ |
| $V_{L,v}$ | Vascular (serum) fraction liver | 0.085 | unity |
| $V_{S,v}$ | Vascular (serum) fraction spleen | 0.12 | unity |
| $V_{K,v}$ | Vascular (serum) fraction kidney | 0.055 | unity |
| $V_{L,int}$ | Interstitial fraction liver | 0.2 | unity |
| $V_{S,int}$ | Interstitial fraction spleen | 0.2 | unity |
| $V_{K,int}$ | Interstitial fraction kidney | 0.15 | unity |
| $F_{L}$ | Serum flow liver arterial | $0.065\cdot F$ | $L\cdot\min^{-1}$ |
| $F_{S}$ | Serum flow spleen | $0.03\cdot F$ | $L\cdot\min^{-1}$ |
| $F_{K}$ | Serum flow kidney | $0.19\cdot F$ | $L\cdot\min^{-1}$ |
| $\varphi$ | Ratio of sieving coefficients | ${\Theta_{\mathrm{DOTATATE}}}/{\theta_{\mathrm{Cr}-51-\mathrm{EDTA}}}=0.66$ | unity |
| $F_{\mathrm{fil}}$ | Filtration | $\mathrm{GF}R_{\mathrm{measured}}\cdot\varphi$ | $L\cdot\min^{-1}$ |
| $F_{\mathrm{ex}}$ | Excretion | $F_{\mathrm{fill}}\cdot f_{\mathrm{ex}}$ | $L\cdot\min^{-1}$ |
| $f_{\mathrm{ex}}$ | Excretion/Filtration | 0.98 | unity |
| $K_{L}$ | Permeability surface area product per unit mass for liver | $K_{\mathrm{MUS}}\cdot100$ | $\mathrm{mL}\cdot\min^{-1}\cdot g^{-1}$ |
| $K_{S}$ | Permeability surface area product per unit mass for spleen | $K_{L}$ (due to similar capillary structure) | $\mathrm{mL}\cdot\min^{-1}\cdot g^{-1}$ |
| $\left[ R_{L,0} \right]$ | Receptor density liver | fitted | $nmol\cdot L^{-1}$ |
| $\left[ R_{S,0} \right]$ | Receptor density spleen | fitted | $nmol\cdot L^{-1}$ |
| $\left[ R_{K,0} \right]$ | Receptor density kidneys | fitted | $nmol\cdot L^{-1}$ |
| $\lambda_{int,L}$ | Internalization rate sst2 liver | $\lambda_{int,K}$ | $\min^{-1}$ |
| $\lambda_{int,S}$ | Internalization rate sst2 spleen | $\lambda_{int,K}$ | $\min^{-1}$ |
| $\lambda_{int,K}$ | Internalization rate sst2 kidney | fitted | $\min^{-1}$ |
| $\lambda_{deg, L}$ | Degradation rate liver | $\lambda_{deg, NT}$ | $\min^{-1}$ |
| $\lambda_{deg, S}$ | Degradation rate spleen | $\lambda_{deg, NT}$ | $\min^{-1}$ |
| $\lambda_{deg, K}$ | Degradation rate kidney | $\lambda_{deg, NT}$ | $\min^{-1}$ |
| **Other Organs** | | | |
| $V_{PRO,total}$  $V_{UT,total}$ | Volume total prostate  Volume total uterus | $0.016\cdot\mathrm{BW}/{71}$  $0.080\cdot\mathrm{BW}/{71}$ | $L$ |
| $V_{LU,total}$ | Volume total lungs | $1\cdot\mathrm{BW}/{71}$ | $L$ |
| $V_{AD,total}$ | Volume total adrenals | $0.014\cdot\mathrm{BW}/{71}$ | $L$ |
| $V_{MUS,total}$ | Volume total muscles | $30.078\cdot\mathrm{BW}/{71}$ | $L$ |
| $V_{GI,total}$ | Volume total GI + pancreas | $\left( 0.385+0.548+0.104+0.105 \right)\cdot\mathrm{BW}/{71}$ | $L$ |
| $V_{SKIN,total}$ | Volume total skin | $3.408\cdot\mathrm{BW}/{71}$ | $L$ |
| $V_{ADI,total}$ | Volume total adipose tissue | $13.465\cdot\mathrm{BW}/{71}$ | $L$ |
| $V_{RM,total}$ | Volume total red marrow | $1.1\cdot\mathrm{BW}/{71}$ | $L$ |
| $V_{BONE,total}$ | Volume total bone without red marrow | $10.165\cdot\mathrm{BW}/{71}-V_{RM,total}$ | $L$ |
| $V_{HRT,total}$ | Volume total heart | $0.341\cdot\mathrm{BW}/{71}$ | $L$ |
| $V_{BR,total}$ | Volume total brain | $1.45\cdot\mathrm{BW}/{71}$ | $L$ |
| $V_{\mathrm{BW}}$ | Volume of total body based on $\mathrm{BW}$ | $1 mL \cong1 g$ | $L$ |
| $V_{REST,total}$ | Volume of rest body. $i=$ all organs except tumour | $V_{\mathrm{BW}}-\sum_{i} V_{i,\mathrm{total}}$ | $L$ |
| $V_{PRO,v}$  $V_{UT,v}$ | Vascular fraction prostate/uterus | $0.04\cdot\left( 1-H \right)\cdot V_{PRO,total}$  $0.07\cdot\left( 1-H \right)\cdot V_{UT,total}$ | $L$ |
| $V_{LU,v}$ | Vascular (serum) volume lungs | $0.105\cdot V_{P}$ | $L$ |
| $V_{AD,v}$ | Vascular (serum) volume adrenals | $0.03\cdot\left( 1-H \right)\cdot V_{AD,total}$ | $L$ |
| $V_{MUS,v}$ | Vascular (serum) volume muscles | $0.14\cdot V_{p}$ | $L$ |
| $V_{GI,v}$ | Vascular (serum) volume GI + pancreas | $0.076\cdot V_{p}$ | $L$ |
| $V_{SKIN,v}$ | Vascular (serum) volume skin | $0.03\cdot V_{p}$ | $L$ |
| $V_{ADI,v}$ | Vascular (serum) volume adipose tissue | $0.05\cdot V_{p}$ | $L$ |
| $V_{RM,v}$ | Vascular (serum) volume red marrow | $0.04\cdot V_{p}$ | $L$ |
| $V_{BONE,v}$ | Vascular (serum) volume bone without red marrow | $0.07\cdot V_{p}-V_{\mathrm{RM}}$ | $L$ |
| $V_{HRT,v}$ | Vascular (serum) volume heart | $0.01\cdot V_{p}$ | $L$ |
| $V_{BR,v}$ | Vascular (serum) volume brain | $0.012\cdot V_{p}$ | $L$ |
| $V_{REST,v}$ | Vascular (serum) volume rests $i=$ all organs except tumour | $V_{p}-\sum_{i} V_{i,v}$ | $L$ |
| $V_{\mathrm{ART}}$ | Arterial serum plus ½ serum content of heart | $0.06\cdot V_{p}+0.045\cdot V_{p}$ | $L$ |
| $V_{\mathrm{VENES}}$ | Venous serum plus ½ serum content of heart | $0.18\cdot V_{p}+0.045\cdot V_{p}$ | $L$ |
| $V_{PRO,int}$  $V_{UT,int}$ | Interstitial volume prostate/uterus | $0.25\cdot V_{PRO,total}$  $0.5\cdot V_{UT,total}$ | $L$ |
| $V_{LU,int}$ | Interstitial volume lungs | $V_{LU,v}\cdot\alpha_{\mathrm{LU}}$ | $L$ |
| $V_{AD,int}$ | Interstitial volume adrenals (the value for salivary glands is used) | $0.24\cdot V_{AD,total}$ | $L$ |
| $V_{MUS,int}$ | Interstitial volume muscles | $V_{MUS,v}\cdot\alpha_{\mathrm{MUS}}$ | $L$ |
| $V_{GI,int}$ | Interstitial volume GI + pancreas | $V_{GI,v}\cdot\alpha_{\mathrm{GI}}$ | $L$ |
| $V_{SKIN,int}$ | Interstitial volume skin | $V_{SKIN,v}\cdot\alpha_{\mathrm{SKIN}}$ | $L$ |
| $V_{ADI,int}$ | Interstitial volume adipose tissue | $V_{ADI,v}\cdot\alpha_{\mathrm{ADI}}$ | $L$ |
| $V_{RM,int}$ | Interstitial volume red marrow | $V_{RM,v}\cdot\alpha_{\mathrm{RM}}$ | $L$ |
| $V_{BONE,int}$ | Interstitial volume bone without red marrow | $V_{BONE,v}\cdot\alpha_{\mathrm{BONE}}$ | $L$ |
| $V_{HRT,int}$ | Interstitial volume heart | $V_{HRT,v}\cdot\alpha_{\mathrm{HRT}}$ | $L$ |
| $V_{REST,int}$ | Interstitial volume of rest body | $V_{REST,v}\cdot\alpha_{\mathrm{REST}}$ | $L$ |
| $\alpha_{\mathrm{MUS}}$ | Ratio interstitial to vascular volume average man of muscle | ${V_{MUS,int}}/{V_{MUS,v}}=5.9$ | unity |
| $\alpha_{\mathrm{GI}}$ | Ratio interstitial to vascular volume average man of GI | ${V_{GI,int}}/{V_{GI,v}}=8.8$ | unity |
| $\alpha_{\mathrm{SKIN}}$ | Ratio interstitial to vascular volume average man of skin | ${V_{SKIN,int}}/{V_{SKIN,v}}=8.9$ | unity |
| $\alpha_{\mathrm{ADI}}$ | Ratio interstitial to vascular volume average man of adipose tissue | ${V_{GI,int}}/{V_{ADI,v}}=15.5$ | unity |
| $\alpha_{\mathrm{RM}}$ | Ratio interstitial to vascular volume average man of red marrow | ${V_{RM,int}}/{V_{RM,v}}=3.7$ | unity |
| $\alpha_{\mathrm{HRT}}$ | Ratio interstitial to vascular volume average man of heart | ${V_{HRT,int}}/{V_{HRT,v}}=3.7$ | unity |
| $\alpha_{\mathrm{LU}}$ | Ratio interstitial to vascular volume average man of lungs | ${V_{LU,int}}/{V_{LU,v}}=5.5$ | unity |
| $\alpha_{\mathrm{BONE}}$ | Ratio interstitial to vascular volume average man of bone without red marrow | ${V_{BONE,int}}/{V_{BONE,v}}=9.3$ | unity |
| $\alpha_{\mathrm{REST}}$ | Ratio interstitial to vascular volume average man of rest of the body | ${V_{REST,int}}/{V_{REST,v}}=3.7$ | unity |
| $f_{\mathrm{PRO}}$ |  | 0.18 | $\mathrm{mL}\cdot\min^{-1}\cdot g^{-1}$ |
| $f_{\mathrm{UT}}$ |  | 1 | $\mathrm{mL}\cdot\min^{-1}\cdot g^{-1}$ |
| $f_{\mathrm{AD}}$ |  | 6 | $\mathrm{mL}\cdot\min^{-1}\cdot g^{-1}$ |
| $F_{\mathrm{PRO}}$  $F_{\mathrm{UT}}$ | Total serum flow to prostate/uterus | $f_{\mathrm{PRO}}\cdot\left( 1-H \right)\cdot V_{PRO,total}$  $f_{\mathrm{UT}}\cdot\left( 1-H \right)\cdot V_{UT,total}$ | $\mathrm{mL}\cdot\min^{-1}$ |
| $F_{\mathrm{LU}}$ | Total serum flow to lungs | $F$ | $\mathrm{mL}\cdot\min^{-1}$ |
| $F_{\mathrm{AD}}$ | Total serum flow to adrenals | $f_{\mathrm{AD}}\cdot\left( 1-H \right)\cdot V_{AD,total}$ | $\mathrm{mL}\cdot\min^{-1}$ |
| $F_{\mathrm{MUS}}$ | Total serum flow to muscles | $0.17\cdot F$ | $\mathrm{mL}\cdot\min^{-1}$ |
| $F_{\mathrm{GI}}$ | Total serum flow to GI + pancreas | $0.16\cdot F$ | $\mathrm{mL}\cdot\min^{-1}$ |
| $F_{\mathrm{SKIN}}$ | Total serum flow to skin | $0.05\cdot F$ | $\mathrm{mL}\cdot\min^{-1}$ |
| $F_{\mathrm{ADI}}$ | Total serum flow to adipose | $0.05\cdot F$ | $\mathrm{mL}\cdot\min^{-1}$ |
| $F_{\mathrm{RM}}$ | Total serum flow to red marrow (RM) | $0.03\cdot F$ | $\mathrm{mL}\cdot\min^{-1}$ |
| $F_{\mathrm{BONE}}$ | Total serum flow to bone (without RM) | $0.05\cdot F$ | $\mathrm{mL}\cdot\min^{-1}$ |
| $F_{\mathrm{HRT}}$ | Total serum flow to heart | $0.04\cdot F$ | $\mathrm{mL}\cdot\min^{-1}$ |
| $F_{\mathrm{BR}}$ | Total serum flow to brain | $0.12\cdot F$ | $\mathrm{mL}\cdot\min^{-1}$ |
| $F_{\mathrm{REST}}$ | $i=$ all organs except tumour | $F-\sum_{i} F_{i}$ | $\mathrm{mL}\cdot\min^{-1}$ |
| $F_{\mathrm{TOTAL}}$ |  | $F+F_{\mathrm{TU}}$ | $\mathrm{mL}\cdot\min^{-1}$ |
| $PS_{i}$ | Permeability surface area product | $k_{i}\cdot V_{i,\mathrm{total}}$ | $\mathrm{mL}\cdot\min^{-1}$ |
| $k_{\mathrm{PRO}}$  $k_{\mathrm{UT}}$ | Permeability surface area product per unit mass (scaled for molecule size of DOTATAE) for prostate/uterus | 0.1  0.2 | $\mathrm{mL}\cdot\min^{-1}\cdot g^{-1}$ |
| $k_{\mathrm{LU}}$ | Permeability surface area product per unit mass for lungs | $k_{\mathrm{MUS}}\cdot100$ | $\mathrm{mL}\cdot\min^{-1}\cdot g^{-1}$ |
| $k_{\mathrm{AD}}$ | Permeability surface area product per unit mass for lungs | $k_{\mathrm{MUS}}\cdot100$ (assumed to be very high as for salivary glands) | $\mathrm{mL}\cdot\min^{-1}\cdot g^{-1}$ |
| $k_{\mathrm{MUS}}$ | Permeability surface area product per unit mass for lungs | 0.02 | $\mathrm{mL}\cdot\min^{-1}\cdot g^{-1}$ |
| $k_{\mathrm{GI}}$ | Permeability surface area product per unit mass for lungs | 0.02 (assumed to similar to muscle) | $\mathrm{mL}\cdot\min^{-1}\cdot g^{-1}$ |
| $k_{\mathrm{SKIN}}$ | Permeability surface area product per unit mass for lungs | 0.02 (assumed to similar to muscle) | $\mathrm{mL}\cdot\min^{-1}\cdot g^{-1}$ |
| $k_{\mathrm{ADI}}$ | Permeability surface area product per unit mass for lungs | 0.02 (assumed to similar to muscle) | $\mathrm{mL}\cdot\min^{-1}\cdot g^{-1}$ |
| $k_{RM}$ | Permeability surface area product per unit mass for lungs | 0.02 (assumed to similar to muscle) | $\mathrm{mL}\cdot\min^{-1}\cdot g^{-1}$ |
| $k_{\mathrm{HRT}}$ | Permeability surface area product per unit mass for lungs | 0.02 (assumed to similar to muscle) | $\mathrm{mL}\cdot\min^{-1}\cdot g^{-1}$ |
| $k_{\mathrm{BONE}}$ | Permeability surface area product per unit mass for lungs | 0.02 (assumed to similar to muscle) | $\mathrm{mL}\cdot\min^{-1}\cdot g^{-1}$ |
| $k_{\mathrm{REST}}$ | Permeability surface area product per unit mass for lungs | 0.02 (assumed to similar to muscle) | $\mathrm{mL}\cdot\min^{-1}\cdot g^{-1}$ |
| $\left[ R_{\mathrm{PRO},0} \right]$  $\left[ R_{\mathrm{UT},0} \right]$ | Receptor density prostate/uterus based on sst2 density ratio | $\left[ R_{K,0} \right]\cdot0.26$  $\left[ R_{K,0} \right]\cdot0.092$ | $nmol\cdot L^{-1}$ |
| $\left[ R_{\mathrm{AD},0} \right]$ | Receptor density adrenal based on sst2 density ratio | $\left[ R_{K,0} \right]\cdot1.65$ | $nmol\cdot L^{-1}$ |
| $\left[ R_{\mathrm{MUS},0} \right]$ | Receptor density muscles based on sst2 density ratio | $\left[ R_{K,0} \right]\cdot0.0056$ | $nmol\cdot L^{-1}$ |
| $\left[ R_{\mathrm{GI},0} \right]$ | Receptor density GI + pancreas based on sst2 density ratio | $\left[ R_{K,0} \right]\cdot0.16$ | $nmol\cdot L^{-1}$ |
| $\left[ R_{\mathrm{RM},0} \right]$ | Receptor density RM based on sst2 density ratio | $\left[ R_{K,0} \right]\cdot0.028$ | $nmol\cdot L^{-1}$ |
| $\left[ R_{\mathrm{REST},0} \right]$ | Receptor density rest based on sst2 density ratio | fitted | $nmol\cdot L^{-1}$ |
| $\lambda_{int,NT}$ | Internalization rate for sst2 normal tissue | $\lambda_{int,K}$ | $\min^{-1}$ |
| $\lambda_{deg,NT}$ | Degradation rate for sst2 cells normal tissue | fitted | $\min^{-1}$ |
| $R$ | Receptor free |  | $\mathrm{nmol}$ |
| $R_{i,0}$ | Receptor total number of organs $i$ | $\left[ R_{i,0} \right]\cdot V_{i,\mathrm{total}}$ | $\mathrm{nmol}$ |
| $\left[ R_{i,0} \right]$ | Receptor density of organs $i$ |  | $nmol\cdot L^{-1}$ |
| $RP_{i}$ | Peptide bound |  | $\mathrm{nmol}$ |
| $\mathrm{PPR}$ | Peptide bound to serum protein |  | $\mathrm{nmol}$ |
| $k_{on,Alb}$ | Binding rate peptide to serum | fitted | $\min^{-1}$ |
| $P_{\mathrm{intern}}$ | Peptide internalised |  | $\mathrm{nmol}$ |
| $P_{i,v}$ | Peptide free vascular |  | $\mathrm{nmol}$ |
| $P_{i,int}$ | Peptide free interstitial |  | $\mathrm{nmol}$ |
| $P_{K,intra}$ | Peptide intracellular kidneys |  | $\mathrm{nmol}$ |

Table S2. Comparison of the parameter set of the fitted parameters between setting I and II. Set of the fitted parameters of setting I was adapted from Hardiansyah et al. [2] and setting II were fitted all the unknown parameters plus the prior knowledge of the tumour blood flow rate [1, 3-5].

| **Parameter sets** | **Setting I [2]** | **Setting II** |
| --- | --- | --- |
| $\left[ R_{K\text{,0}} \right]\left( \text{nmol/L} \right)$ | fitted | fitted |
| $\left[ R_{L\text{,0}} \right] \left( \text{nmol/L} \right)$ | fitted | fitted |
| $\left[ R_{S\text{,0}} \right] \left( \text{nmol/L} \right)$ | fitted | fitted |
| $\left[ R_{\mathrm{TU}\text{,0}} \right] \left( \text{nmol/L} \right)$ | fitted | fitted |
| $\left[ R_{\mathrm{Rest}\text{,0}} \right] \left( \text{nmol/L} \right)$ | fixed to 0.5 | fitted |
| $k_{\mathrm{on}\text{, Alb}} \left( \text{nmol/L} \right)$ | Fitted | fitted |
| $f_{\mathrm{TU}} \left( \mathrm{mL}\cdot\min^{-1}\cdot g^{-1} \right)$ | Fitted | Fixed to 0.9 for meningioma [1, 4]  Fixed to 1.0 for NETs [1, 5] |
| $\lambda_{int, K} \left( \text{min}^{-1} \right)$ | Fixed to 1.7 × 10^-3^ | fitted |
| $\lambda_{int, TU} \left( \text{min}^{-1} \right)$ | Fixed to 1.0 × 10^-3^ | fitted |
| $\lambda_{deg, NT} \left( \text{min}^{-1} \right)$ | fitted | fitted |
| $\lambda_{deg, TU} \left( \text{min}^{-1} \right)$ | Fixed to 1.695 × 10^-4^ | Fitted |
| $a$ (in Eq. 2)  Blood serum  Whole body  Kidney  Spleen  Liver  Tumours | Fitted, all intra-individual variability were described using a single $a$ value in the model for all organs | Fitted  Fitted  Fitted  Fitted  Fitted  Fitted |

Table S3. Reference time-integrated activity coefficients (rTIACs) and standard errors for tumours and investigated organs obtained from all-time-point (ATP) fitting of the PBPK model to all biokinetic data.

| **Patient ID** | **Blood Serum (min)** | **Whole Body (min)** | **Kidney (min)** | **Spleen (min)** | **Liver (min)** | **Tumour (min)** |
| --- | --- | --- | --- | --- | --- | --- |
| 1 | 20 ± 1 | 956 ± 38 | 139 ± 4 | 100 ± 3 | 161 ± 8 | 52 ± 2 |
| 2 | 19 ± 1 | 904 ± 28 | 114 ± 3 | 81 ± 3 | 108 ± 6 | 168 ± 7 |
| 3 | 24 ± 1 | 997 ± 40 | 106 ± 3 | 121 ± 5 | 97 ± 5 | 0.90 ± 0.04 |
| 4 | 18 ± 1 | 1099 ± 43 | 188 ± 6 | 164 ± 6 | 108 ± 6 | 4.7 ± 0.2 |
| 5 | 28 ± 2 | 1151 ± 44 | 144 ± 5 | -* | 194 ± 10 | 49 ± 2 |
| 6 | 28 ± 1 | 1093 ± 34 | 82 ± 2 | 54 ± 2 | 198 ± 10 | 98 ± 5 |
| 7 | 62 ± 3 | 1641 ± 64 | 144 ± 4 | 83 ± 3 | 157 ± 8 | 23 ± 1 |
| 8 | 44 ± 3 | 2237 ± 91 | 211 ± 6 | 226 ± 8 | 331 ± 16 | 7.8 ± 0.4 |

-* splenectomy

Table S4. Performance of single-time-point (STP) dosimetry for tumour and investigated organs on each time-point. The relative deviation (RD) was calculated using Equation (3), and it is relative to the all-time-point (ATP) fitting. The RD standard errors were calculated based on the error propagation [6].

| **ID** | **Time (min)** | **Blood Serum** | | **Whole Body** | | **Kidney** | | **Spleen** | | **Liver** | | **Tumour** | |
| --- | --- | --- | --- | --- | --- | --- | --- | --- | --- | --- | --- | --- | --- |
|  |  | **sTIAC (min)** | **RD** | **sTIAC (min)** | **RD** | **sTIAC (min)** | **RD** | **sTIAC (min)** | **RD** | **sTIAC (min)** | **RD** | **sTIAC (min)** | **RD** |
| 1 | 122.4 | 21 ± 5 | (4 ± 26)% | 1034 ± 197 | (8 ± 21)% | 153 ± 32 | (10 ± 23)% | 103 ± 20 | (3 ± 20)% | 217 ± 44 | (34 ± 28)% | 55 ± 22 | (5 ± 42)% |
|  | 262.2 | 22 ± 5 | (8 ± 26)% | 888 ± 125 | (-7 ± 14)% | 122 ± 20 | (-12 ± 15)% | 91 ± 14 | (-9 ± 15)% | 168 ± 26 | (4 ± 17)% | 56 ± 17 | (7 ± 32)% |
|  | 1286.4 | 21 ± 5 | (7 ± 26)% | 927 ± 80 | (-3 ± 9)% | 130 ± 12 | (-7 ± 9)% | 95 ± 8 | (-5 ± 9)% | 156 ± 13 | (-3 ± 9)% | 56 ± 12 | (7 ± 24)% |
|  | 2749.8 | 21 ± 5 | (5 ± 25)% | 961 ± 55 | (0 ± 7)% | 140 ± 9 | (1 ± 7)% | 103 ± 6 | (3 ± 7)% | 151 ± 8 | (-6 ± 7)% | 56 ± 6 | (7 ± 13)% |
|  | 4254.6 | 20 ± 5 | (-1 ± 25)% | 929 ± 48 | (-3 ± 6)% | 140 ± 10 | (0 ± 7)% | 102 ± 3 | (2 ± 5)% | 129 ± 8 | (-20 ± 6)% | 53 ± 4 | (1 ± 10)% |
| 2 | 125.4 | 19 ± 5 | (4 ± 27)% | 1081 ± 180 | (20 ± 20)% | 108 ± 21 | (-5 ± 19)% | 73 ± 15 | (-10 ± 19)% | 125 ± 31 | (16 ± 29)% | 216 ± 67 | (29 ± 40)% |
|  | 253.2 | 20 ± 4 | (7 ± 23)% | 961 ± 115 | (6 ± 13)% | 98 ± 13 | (-14 ± 12)% | 69 ± 10 | (-15 ± 13)% | 111 ± 19 | (3 ± 18)% | 200 ± 55 | (19 ± 33)% |
|  | 1300.8 | 20 ± 4 | (7 ± 24)% | 966 ± 85 | (7 ± 10)% | 120 ± 13 | (5 ± 12)% | 90 ± 10 | (10 ± 13)% | 113 ± 13 | (5 ± 13)% | 182 ± 32 | (9 ± 20)% |
|  | 2744.4 | 20 ± 4 | (6 ± 22)% | 952 ± 41 | (5 ± 6)% | 117 ± 8 | (2 ± 7)% | 84 ± 4 | (4 ± 7)% | 106 ± 7 | (-2 ± 8)% | 197 ± 19 | (17 ± 13)% |
|  | 4209.6 | 20 ± 4 | (7 ± 23)% | 920 ± 34 | (2 ± 5)% | 119 ± 7 | (4 ± 7)% | 85 ± 4 | (4 ± 6)% | 101 ± 5 | (-6 ± 7)% | 148 ± 13 | (-12 ± 8)% |
| 3 | 161.2 | 25 ± 6 | (4 ± 26)% | 1008 ± 214 | (1 ± 22)% | 111 ± 26 | (5 ± 25)% | 133 ± 33 | (9 ± 27)% | 143 ± 35 | (46 ± 37)% | 1.4 ± 0.4 | (54 ± 48)% |
|  | 257.8 | 26 ± 6 | (8 ± 25)% | 825 ± 145 | (-17 ± 15)% | 78 ± 15 | (-26 ± 14)% | 95 ± 17 | (-22 ± 14)% | 113 ± 20 | (16 ± 21)% | 1.3 ± 0.6 | (45 ± 63)% |
|  | 1396 | 26 ± 6 | (8 ± 25)% | 972 ± 115 | (-2 ± 12)% | 109 ± 16 | (3 ± 15)% | 112 ± 15 | (-8 ± 13)% | 91 ± 12 | (-6 ± 14)% | 0.9 ± 0.2 | (1 ± 19)% |
|  | 2711.8 | 26 ± 6 | (6 ± 25)% | 1033 ± 56 | (4 ± 7)% | 117 ± 8 | (11 ± 8)% | 123 ± 6 | (1 ± 6)% | 90 ± 5 | (-7 ± 7)% | 1.1 ± 0.1 | (17 ± 12)% |
|  | 4154.2 | 26 ± 6 | (9 ± 26)% | 1006 ± 65 | (1 ± 8)% | 98 ± 6 | (-7 ± 7)% | 135 ± 6 | (11 ± 6)% | 78 ± 4 | (-20 ± 6)% | 0.8 ± 0.1 | (-15 ± 9)% |
| 4 | 176.4 | 18 ± 4 | (2 ± 23)% | 864 ± 101 | (-21 ± 10)% | 140 ± 20 | (-26 ± 11)% | 133 ± 18 | (-19 ± 11)% | 122 ± 17 | (13 ± 17)% | 16 ± 5 | (233 ± 103)% |
|  | 291.6 | 18 ± 5 | (1 ± 26)% | 1094 ± 184 | (0 ± 17)% | 175 ± 20 | (-7 ± 11)% | 145 ± 16 | (-12 ± 10)% | 122 ± 15 | (12 ± 15)% | 9 ± 3 | (81 ± 56)% |
|  | 1401 | 17 ± 4 | (-2 ± 22)% | 1141 ± 81 | (4 ± 8)% | 218 ± 13 | (16 ± 8)% | 176 ± 10 | (7 ± 7)% | 107 ± 6 | (-1 ± 8)% | 5 ± 1 | (14 ± 21)% |
|  | 2857.2 | 17 ± 4 | (-3 ± 22)% | 1165 ± 72 | (6 ± 8)% | 208 ± 10 | (10 ± 6)% | 190 ± 12 | (16 ± 8)% | 99 ± 6 | (-8 ± 7)% | 5.3 ± 0.4 | (12 ± 10)% |
|  | 4315.8 | 18 ± 4 | (2 ± 23)% | 1167 ± 136 | (6 ± 13)% | 213 ± 31 | (13 ± 17)% | 185 ± 20 | (13 ± 13)% | 102 ± 15 | (-5 ± 15)% | 3.5 ± 0.4 | (-26 ± 8)% |
| 5 | 199.8 | 24 ± 3 | (-15 ± 12)% | 1124 ± 368 | (-2 ± 32)% | 175 ± 66 | (21 ± 46)% | -* | -* | 244 ± 97 | (26 ± 51)% | 53 ± 22 | (8 ± 46)% |
|  | 271.2 | 24 ± 5 | (-15 ± 18)% | 1125 ± 180 | (-2 ± 16)% | 168 ± 21 | (17 ± 15)% | -* | -* | 247 ± 40 | (28 ± 22)% | 57 ± 15 | (17 ± 31)% |
|  | 1325.4 | 23 ± 5 | (-17 ± 18)% | 1167 ± 99 | (1 ± 9)% | 155 ± 11 | (8 ± 8)% | -* | -* | 195 ± 20 | (1 ± 11)% | 61 ± 10 | (26 ± 21)% |
|  | 2773.8 | 23 ± 5 | (-16 ± 18)% | 1206 ± 87 | (5 ± 9)% | 152 ± 8 | (5 ± 6)% | -* | -* | 192 ± 20 | (-1 ± 11)% | 57 ± 7 | (17 ± 14)% |
|  | 4243.2 | 24 ± 5 | (-14 ± 19)% | 1112 ± 78 | (-3 ± 8)% | 131 ± 7 | (-9 ± 6)% | -* | -* | 186 ± 17 | (-4 ± 10)% | 45 ± 4 | (-7 ± 9)% |
| 6 | 180.6 | 35 ± 7 | (25 ± 26)% | 1363 ± 309 | (25 ± 29)% | 96 ± 19 | (17 ± 24)% | 75 ± 15 | (40 ± 28)% | 314 ± 62 | (59 ± 32)% | 55 ± 20 | (-44 ± 21)% |
|  | 326.4 | 36 ± 7 | (28 ± 24)% | 1307 ± 219 | (20 ± 20)% | 95 ± 15 | (16 ± 19)% | 54 ± 9 | (1 ± 17)% | 287 ± 48 | (45 ± 25)% | 66 ± 20 | (-33 ± 21)% |
|  | 1309.2 | 38 ± 6 | (36 ± 23)% | 1028 ± 64 | (-6 ± 7)% | 84 ± 6 | (3 ± 8)% | 51 ± 5 | (-6 ± 9)% | 175 ± 15 | (-12 ± 9)% | 73 ± 12 | (-26 ± 12)% |
|  | 2776.2 | 37 ± 7 | (33 ± 24)% | 1112 ± 59 | (2 ± 6)% | 86 ± 5 | (6 ± 7)% | 54 ± 3 | (1 ± 7)% | 173 ± 10 | (-12 ± 7)% | 92 ± 8 | (-6 ± 9)% |
|  | 4255.8 | 38 ± 7 | (35 ± 24)% | 1121 ± 57 | (3 ± 6)% | 78 ± 5 | (-4 ± 7)% | 58 ± 3 | (7 ± 7)% | 187 ± 13 | (-5 ± 8)% | 116 ± 11 | (18 ± 13)% |
| 7 | 232.8 | 74 ± 16 | (21 ± 26)% | 1273 ± 231 | (-22 ± 14)% | 138 ± 21 | (-4 ± 15)% | 60 ± 10 | (-28 ± 13)% | 126 ± 28 | (-20 ± 18)% | 15 ± 4 | (-36 ± 17)% |
|  | 283.8 | 75 ± 19 | (22 ± 31)% | 1362 ± 168 | (-17 ± 11)% | 118 ± 16 | (-18 ± 11)% | 53 ± 6 | (-36 ± 7)% | 109 ± 11 | (-31 ± 8)% | 13 ± 2 | (-45 ± 8)% |
|  | 1581 | 61 ± 1 | (0 ± 6)% | 2065 ± 38 | (26 ± 5)% | 143 ± 1 | (0 ± 3)% | 104 ± 2 | (25 ± 5)% | 173 ± 3 | (10 ± 6)% | 12.41 ± 0.01 | (-46 ± 3)% |
|  | 3036.6 | 65 ± 16 | (6 ± 27)% | 1594 ± 94 | (-3 ± 7)% | 142 ± 7 | (-2 ± 5)% | 84 ± 5 | (2 ± 7)% | 144 ± 7 | (-8 ± 7)% | 21 ± 2 | (-9 ± 8)% |
|  | 4315.2 | 70 ± 19 | (13 ± 32)% | 1583 ± 85 | (-4 ± 6)% | 156 ± 7 | (8 ± 6)% | 92 ± 4 | (12 ± 6)% | 164 ± 10 | (4 ± 8)% | 25 ± 2 | (10 ± 9)% |
| 8 | 178.2 | 30 ± 4 | (-31 ± 11)% | 2002 ± 209 | (-10 ± 10)% | 272 ± 31 | (29 ± 15)% | 321 ± 40 | (42 ± 19)% | 437 ± 66 | (32 ± 21)% | 7 ± 2 | (-12 ± 31)% |
|  | 274.8 | 30 ± 5 | (-30 ± 12)% | 1969 ± 315 | (-12 ± 15)% | 257 ± 35 | (22 ± 17)% | 303 ± 54 | (34 ± 24)% | 421 ± 87 | (27 ± 27)% | 7 ± 2 | (-7 ± 24)% |
|  | 1328.4 | 27 ± 4 | (-38 ± 11)% | 2197 ± 190 | (-2 ± 9)% | 228 ± 17 | (8 ± 9)% | 233 ± 22 | (3 ± 10)% | 343 ± 37 | (3 ± 12)% | 8 ± 1 | (-1 ± 18)% |
|  | 2787 | 28 ± 4 | (-36 ± 11)% | 2157 ± 165 | (-4 ± 8)% | 221 ± 13 | (5 ± 7)% | 221 ± 17 | (-2 ± 8)% | 336 ± 35 | (1 ± 12)% | 9 ± 1 | (18 ± 15)% |
|  | 4321.8 | 30 ± 4 | (-31 ± 11)% | 2089 ± 138 | (-7 ± 7)% | 206 ± 13 | (-2 ± 7)% | 209 ± 13 | (-8 ± 7)% | 313 ± 29 | (-5 ± 10)% | 9 ± 1 | (15 ± 20)% |

-* splenectomy

Table S5. Root-mean-square error (RMSE) and mean absolute percentage error (MAPE) at each time point. The uncertainties of RMSE and MAPE were calculated through error propagation .

| **Time point** | **Root-mean-square error (RMSE)** | | | | | | **Mean absolute percentage error (MAPE)** | | | | | |
| --- | --- | --- | --- | --- | --- | --- | --- | --- | --- | --- | --- | --- |
|  | **Serum** | **Whole Body** | **Kidneys** | **Spleen** | **Liver** | **Tumour** | **Serum** | **Whole Body** | **Kidneys** | **Spleen** | **Liver** | **Tumour** |
| T1 | (18 ± 7)% | (17 ± 8)% | (18 ± 8)% | (28 ± 8)% | (35 ± 10)% | (93 ± 33)% | (13 ± 8)% | (14 ± 7)% | (15 ± 9)% | (19 ± 7)% | (31 ± 11)% | (53 ± 18)% |
| T2 | (19 ± 8)% | (13 ± 6)% | (19 ± 6)% | (23 ± 8)% | (26 ± 7)% | (42 ± 16)% | (15 ± 8)% | (10 ± 5)% | (17 ± 5)% | (16 ± 5)% | (21 ± 7)% | (32 ± 13)% |
| T3 | (21 ± 7)% | (11 ± 2)% | (8 ± 3)% | (12 ± 3)% | (7 ± 4)% | (23 ± 5)% | (14 ± 7)% | (6 ± 3)% | (6 ± 3)% | (8 ± 3)% | (5 ± 4)% | (16 ± 6)% |
| T4 | (20 ± 7)% | (4 ± 3)% | (7 ± 2)% | (7 ± 3)% | (7 ± 3)% | (14 ± 4)% | (14 ± 8)% | (4 ± 3)% | (5 ± 2)% | (3 ± 2)% | (6 ± 3)% | (13 ± 4)% |
| T5 | (20 ± 7)% | (4 ± 4)% | (8 ± 4)% | (9 ± 3)% | (11 ± 3)% | (16 ± 5)% | (14 ± 8)% | (3 ± 3)% | (6 ± 3)% | (7 ± 2)% | (9 ± 3)% | (13 ± 4)% |

**Part C: Supplemental Figures**

**
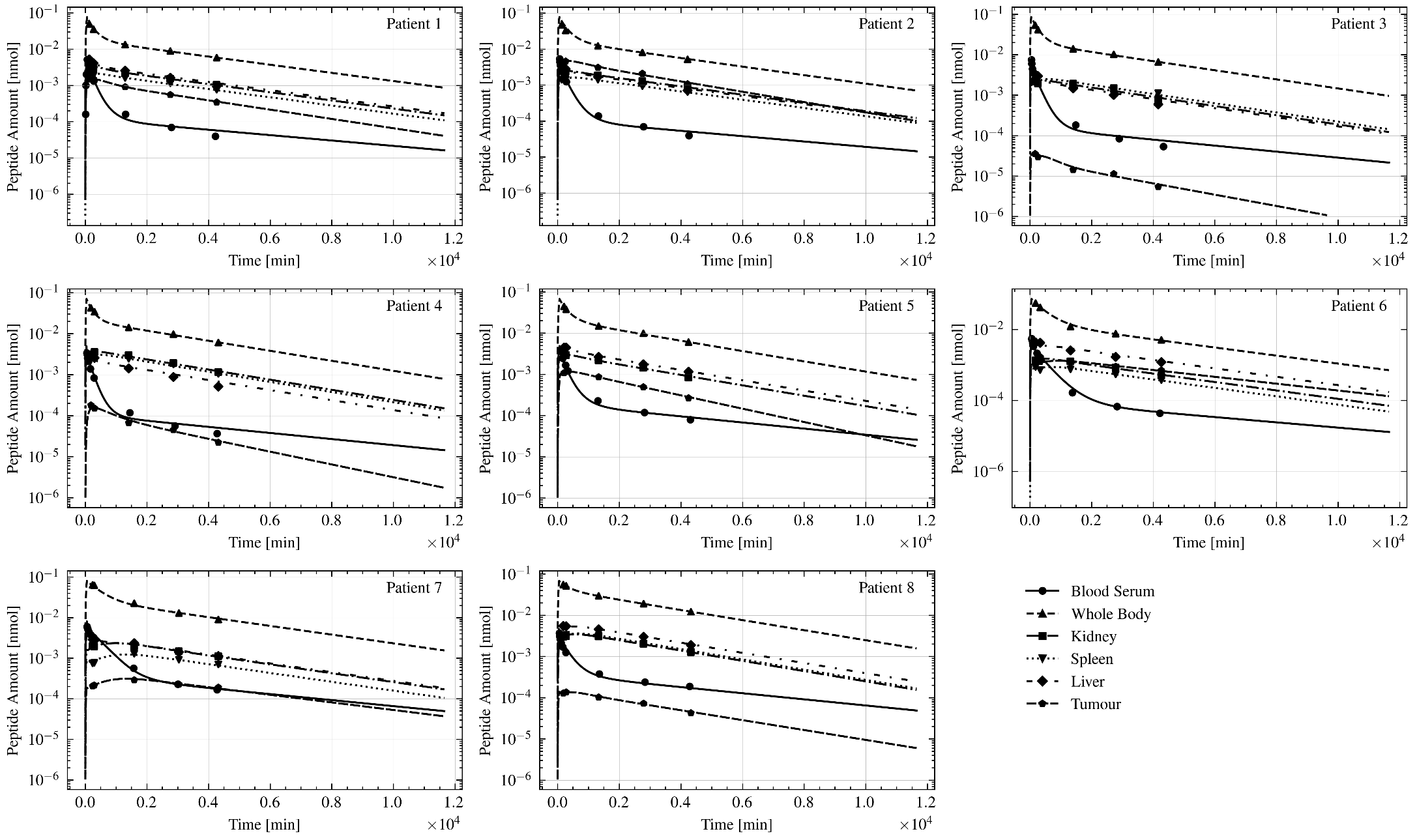
**

Figure S1. The biokinetic curves of [^111^In]In-DOTA-TATE for the tumours and the investigated organs generated from all-time-point (ATP) fitting using the physiologically-based pharmacokinetic (PBPK) model. The $y$-axis is presented in a log-scale.


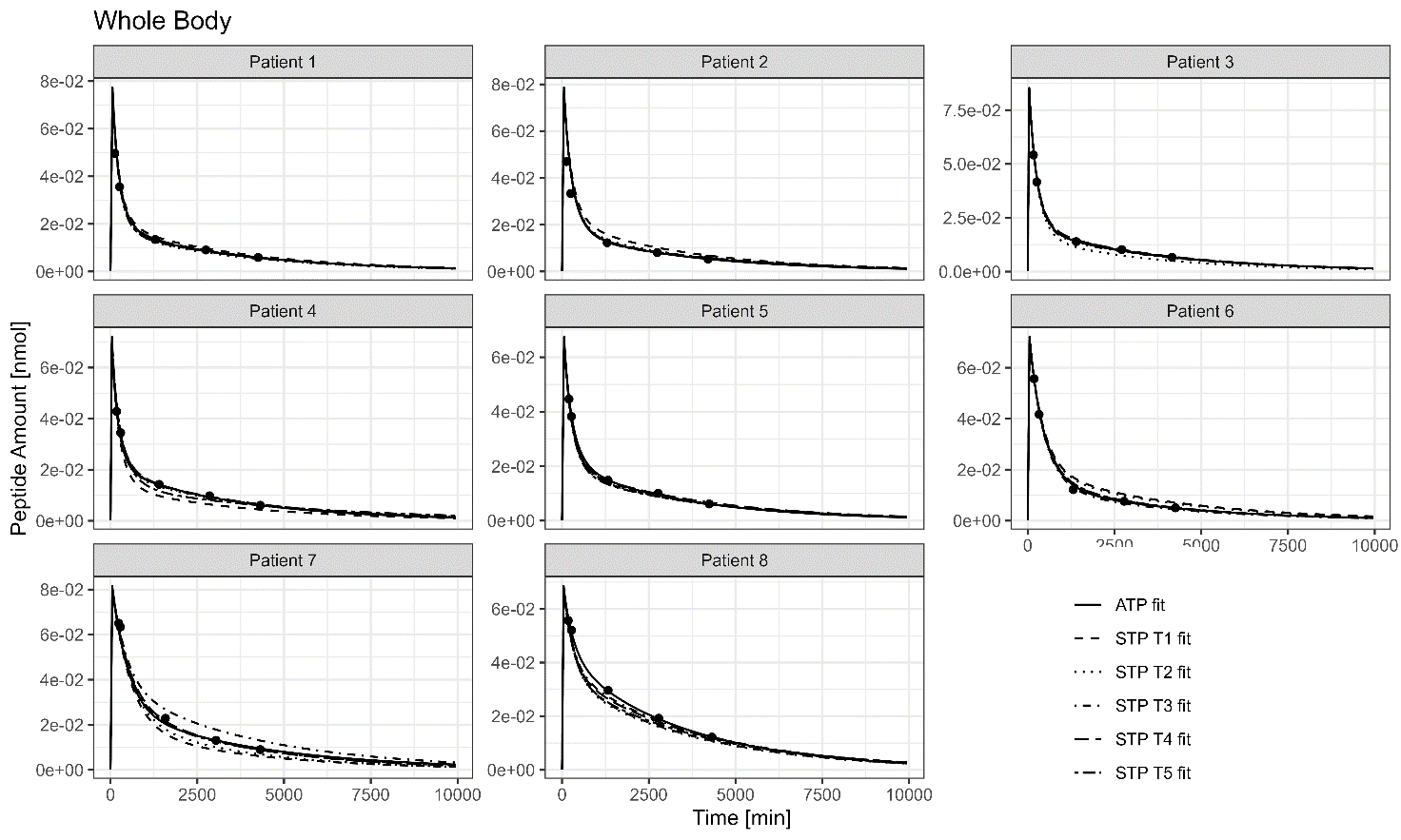


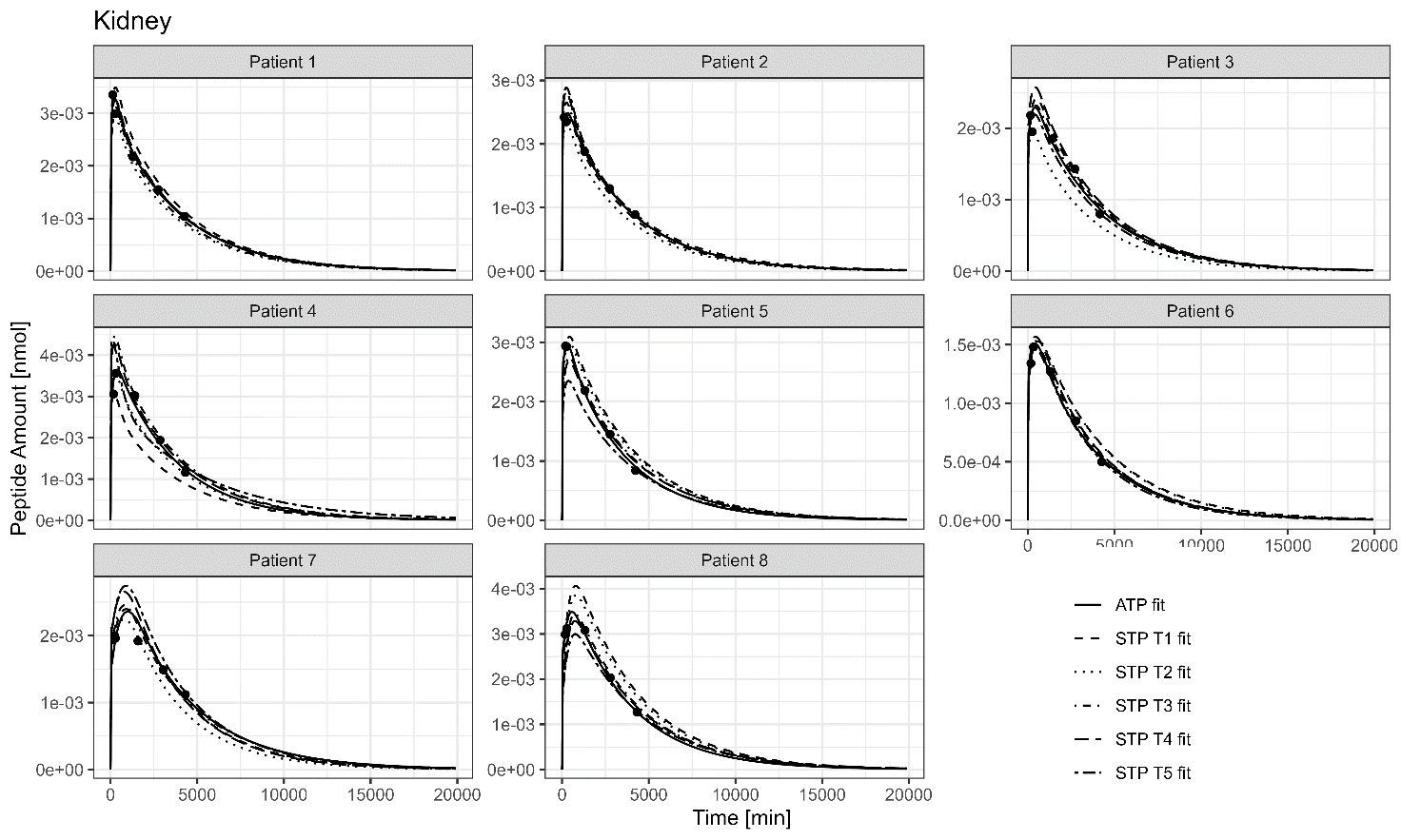


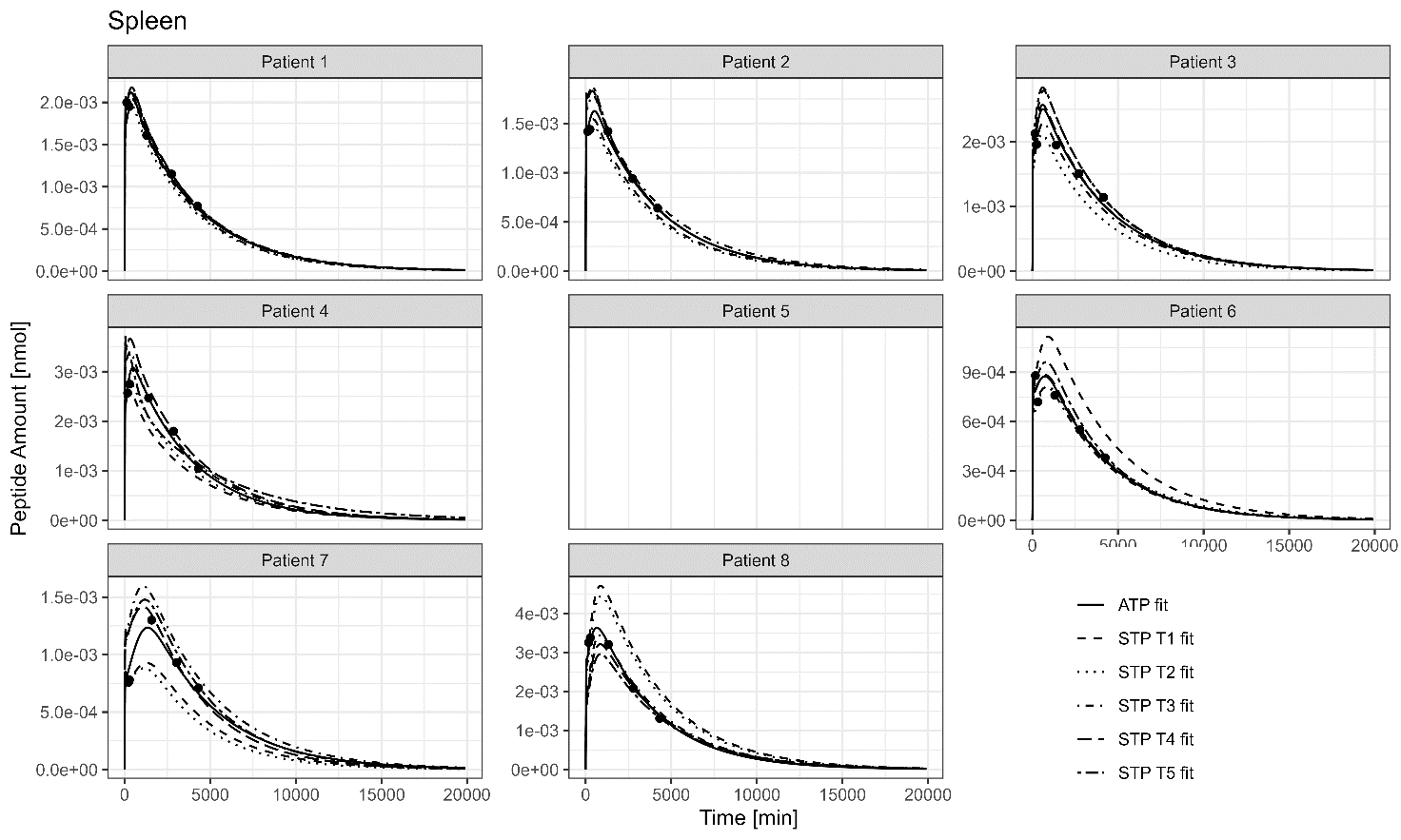


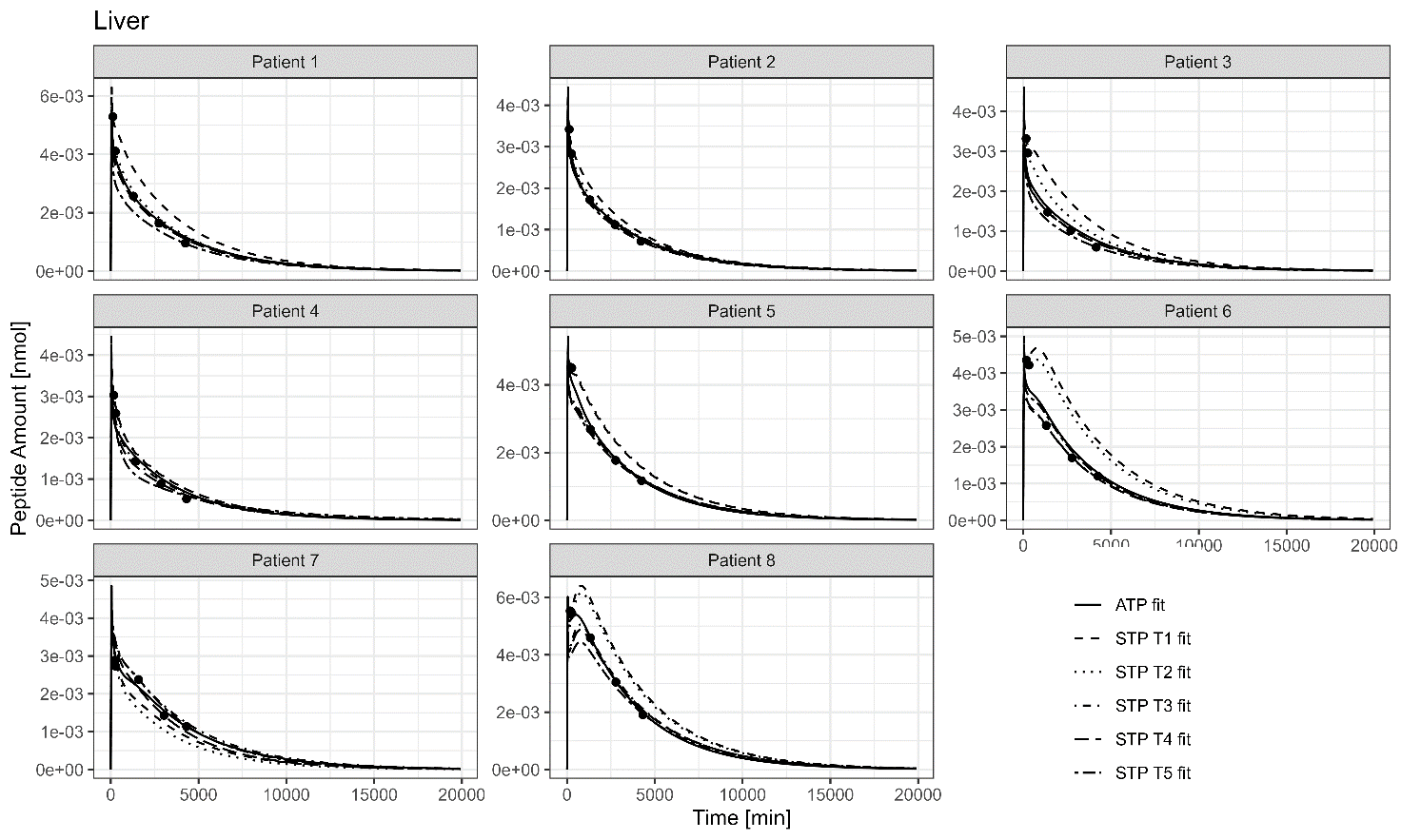


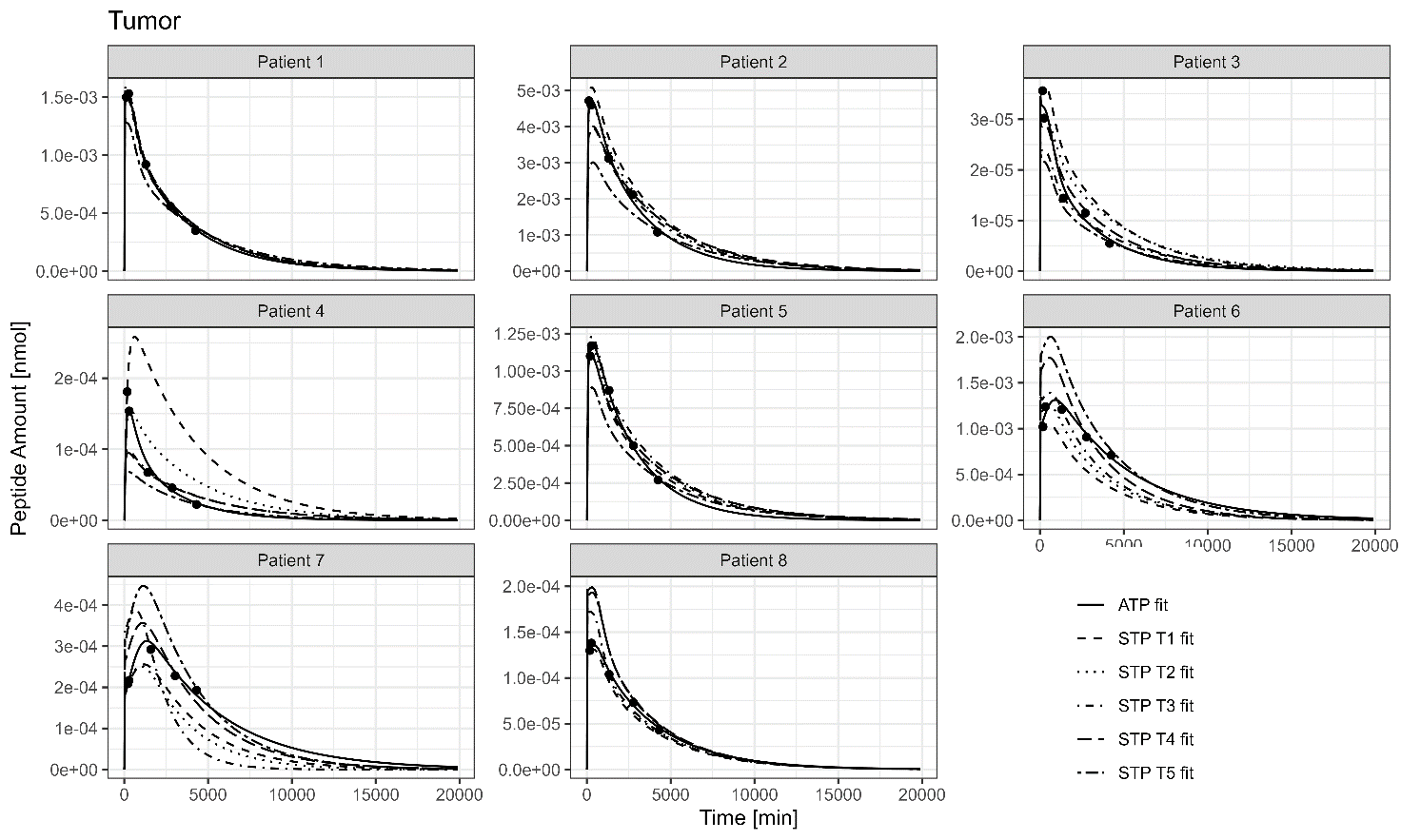


Figure S2. Biokinetic data and fit curves generated using a physiologically-based pharmacokinetic (PBPK) model with nonlinear mixed-effects modelling (NLMEM). The time-activity curves were fitted to all-time-point (ATP) data and single-time-point (STP) imaging data at time points T1 (2 h), T2 (4 h), T3 (24 h), T4 (48 h), and T5 (72 h) after injection. Patient 5 had a splenectomy. Consequently, patient number 5 does not have any data on the spleen. All blood serum data were excluded in STP fitting as performed in the literature [2].


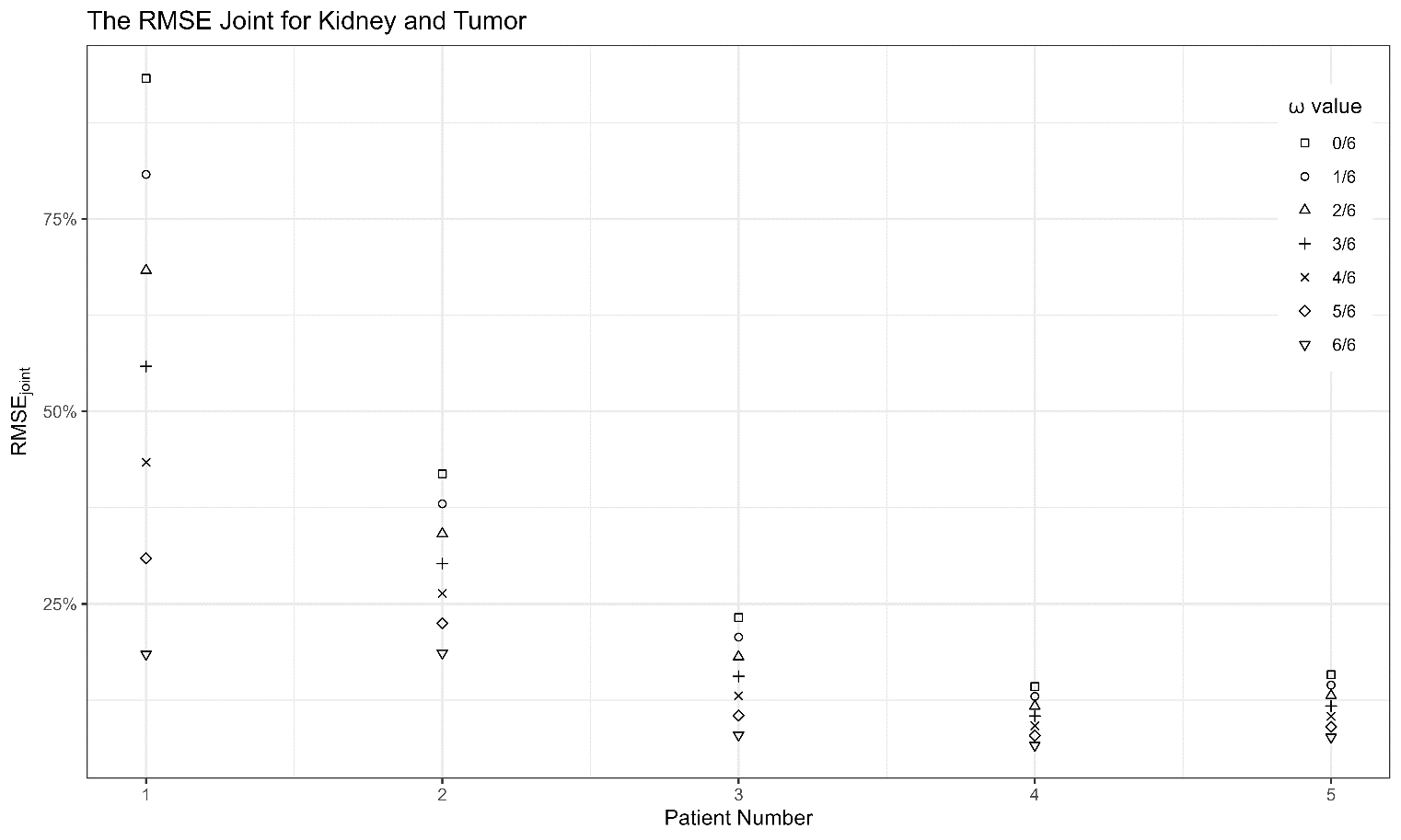


Figure S3. The RMSE_joint_ for single-time-point (STP) imaging dosimetry at various time points was calculated based on Equation (5).


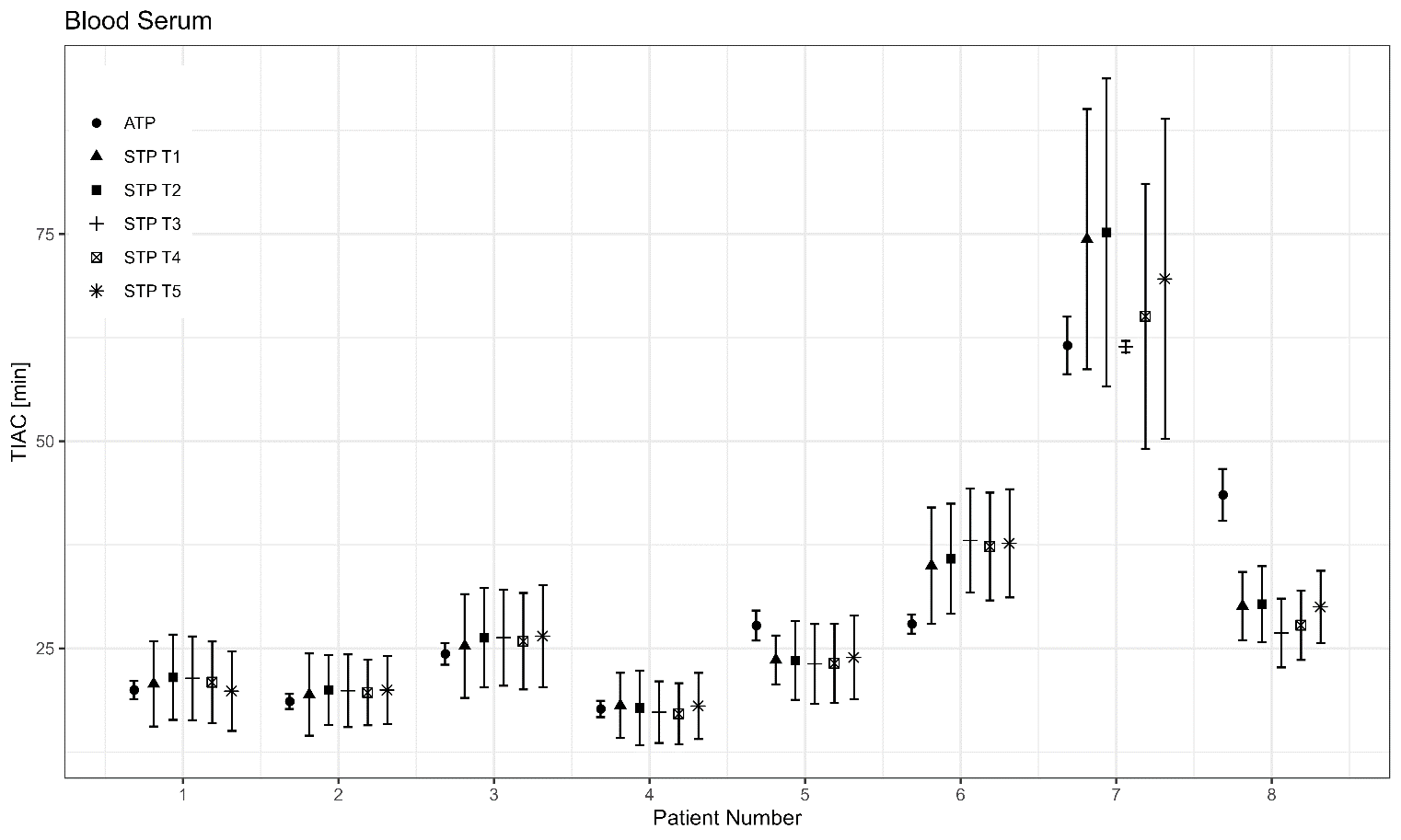

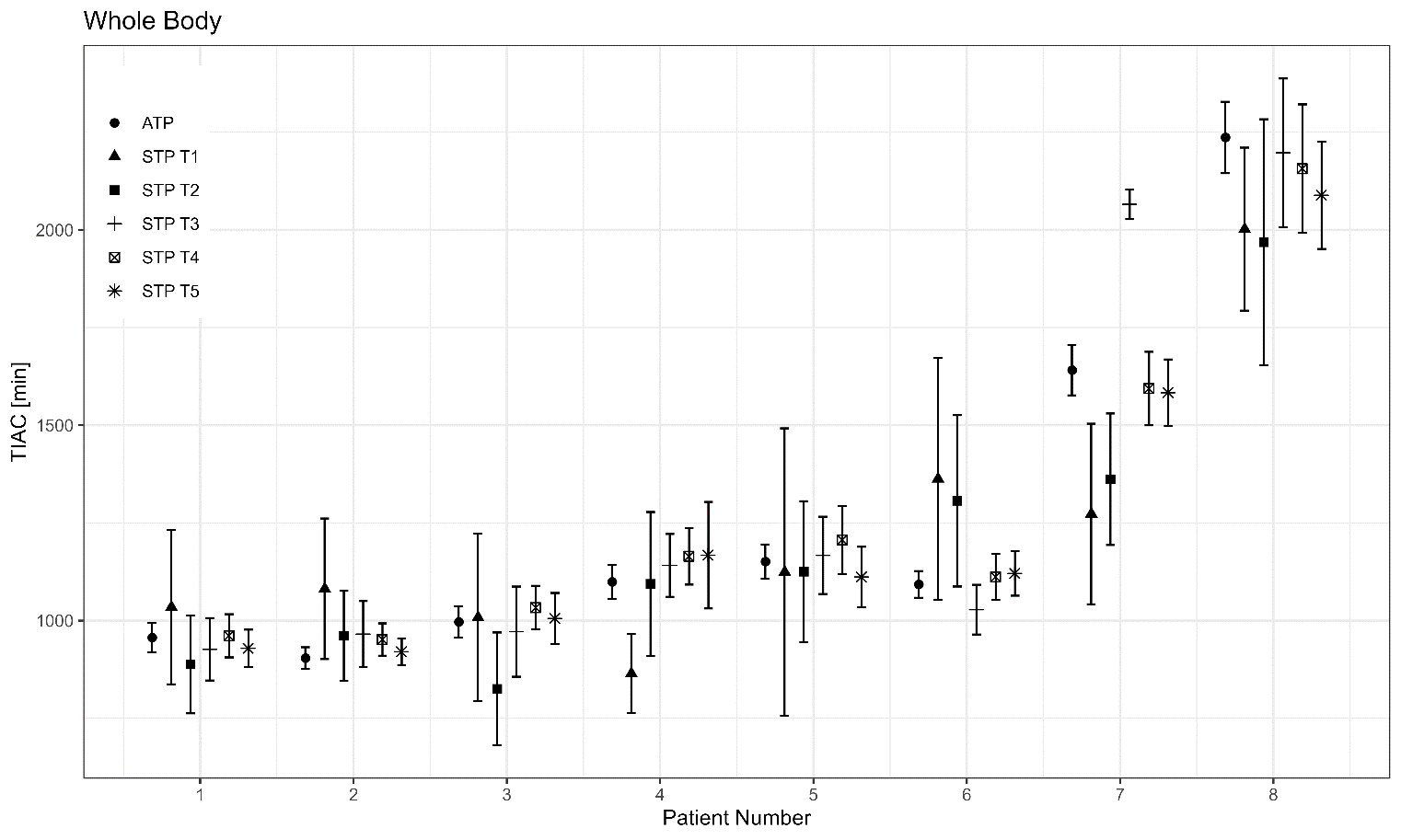

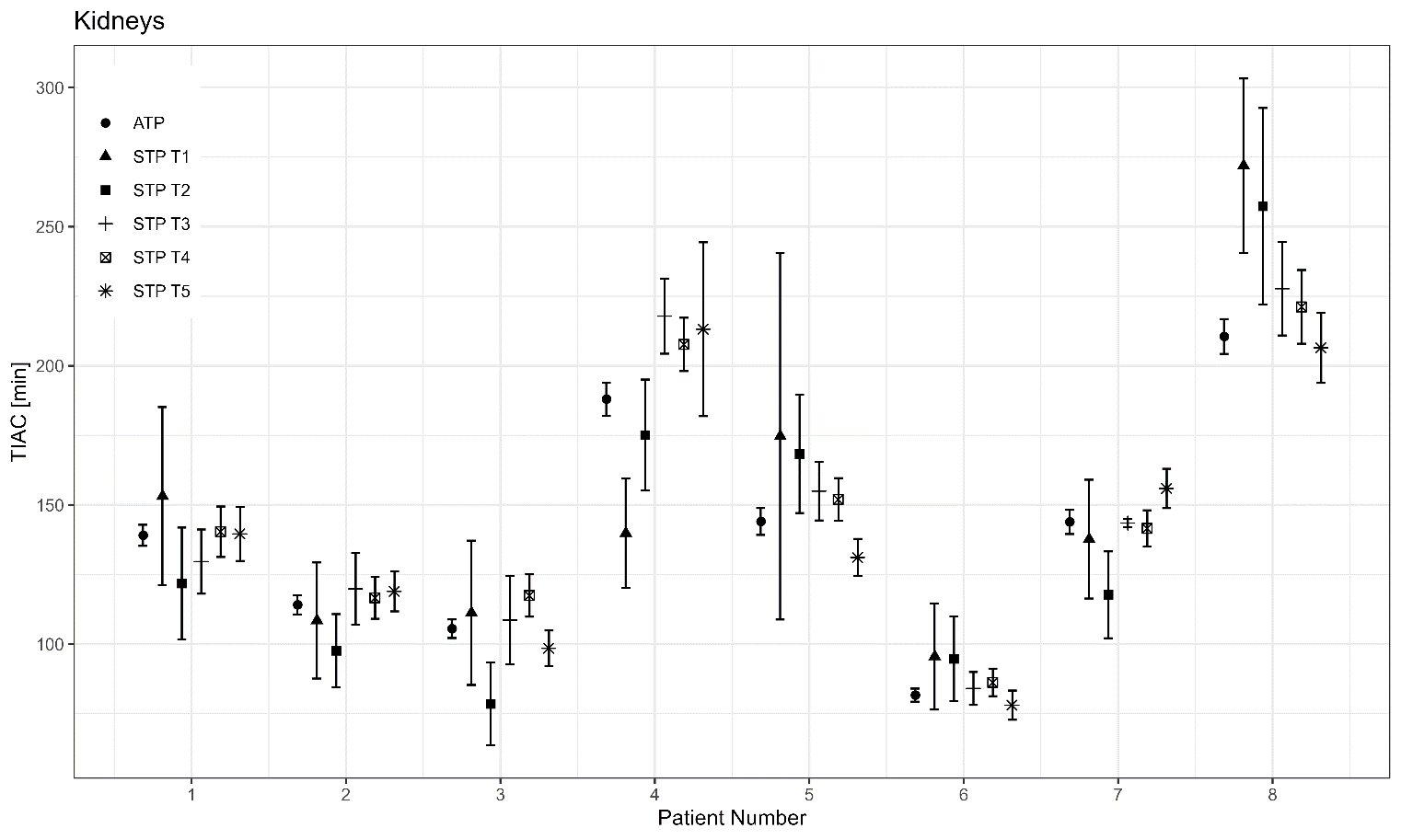


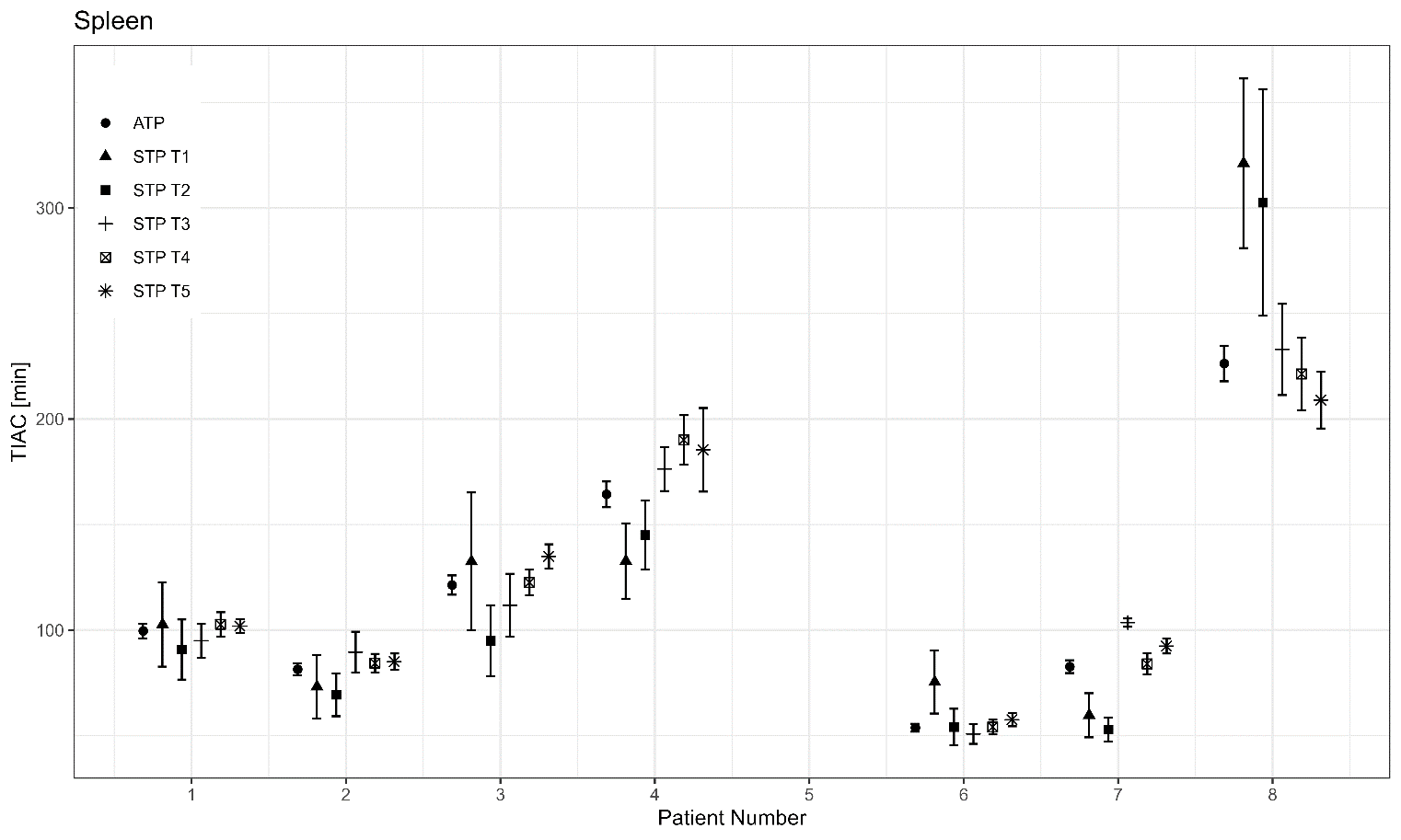


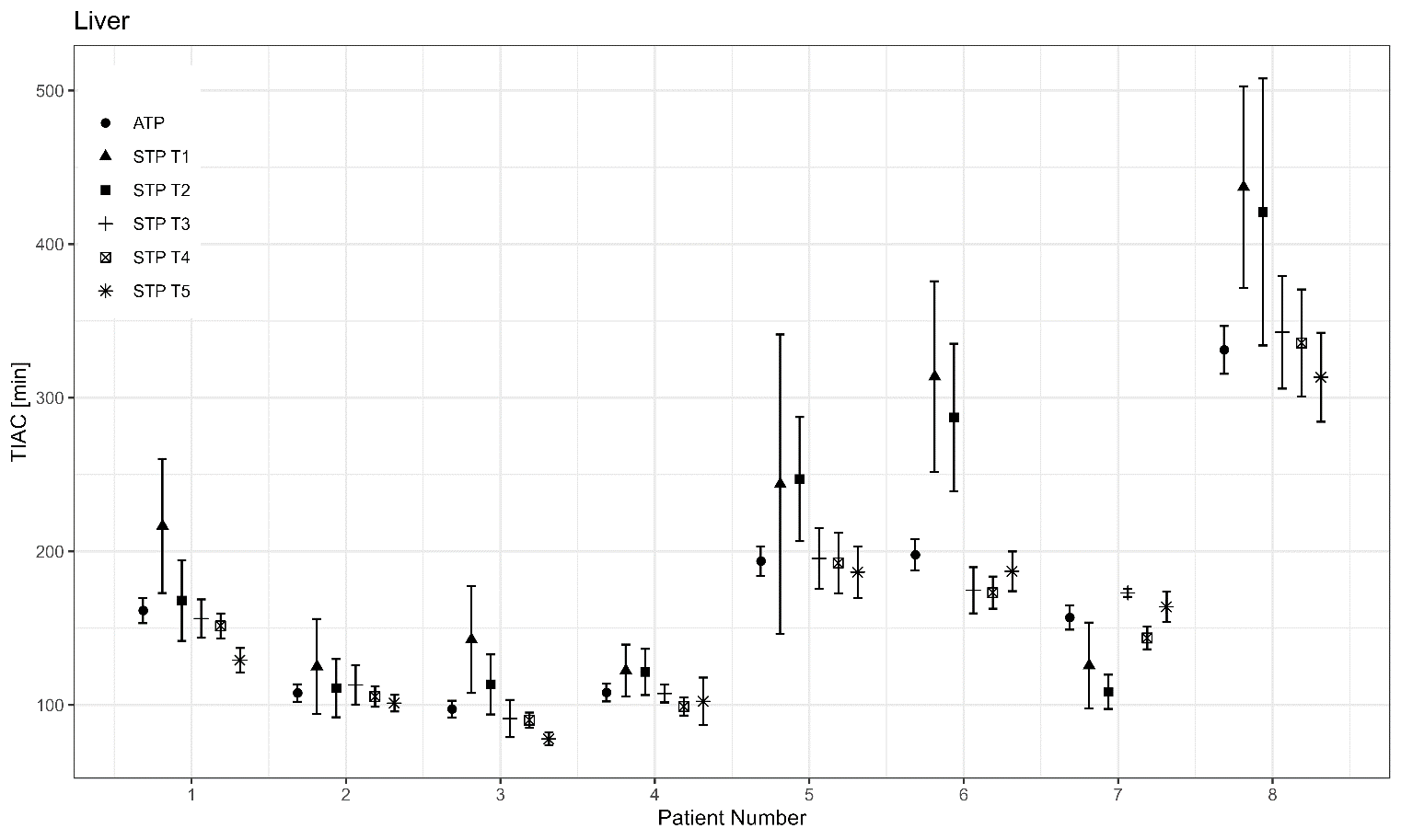


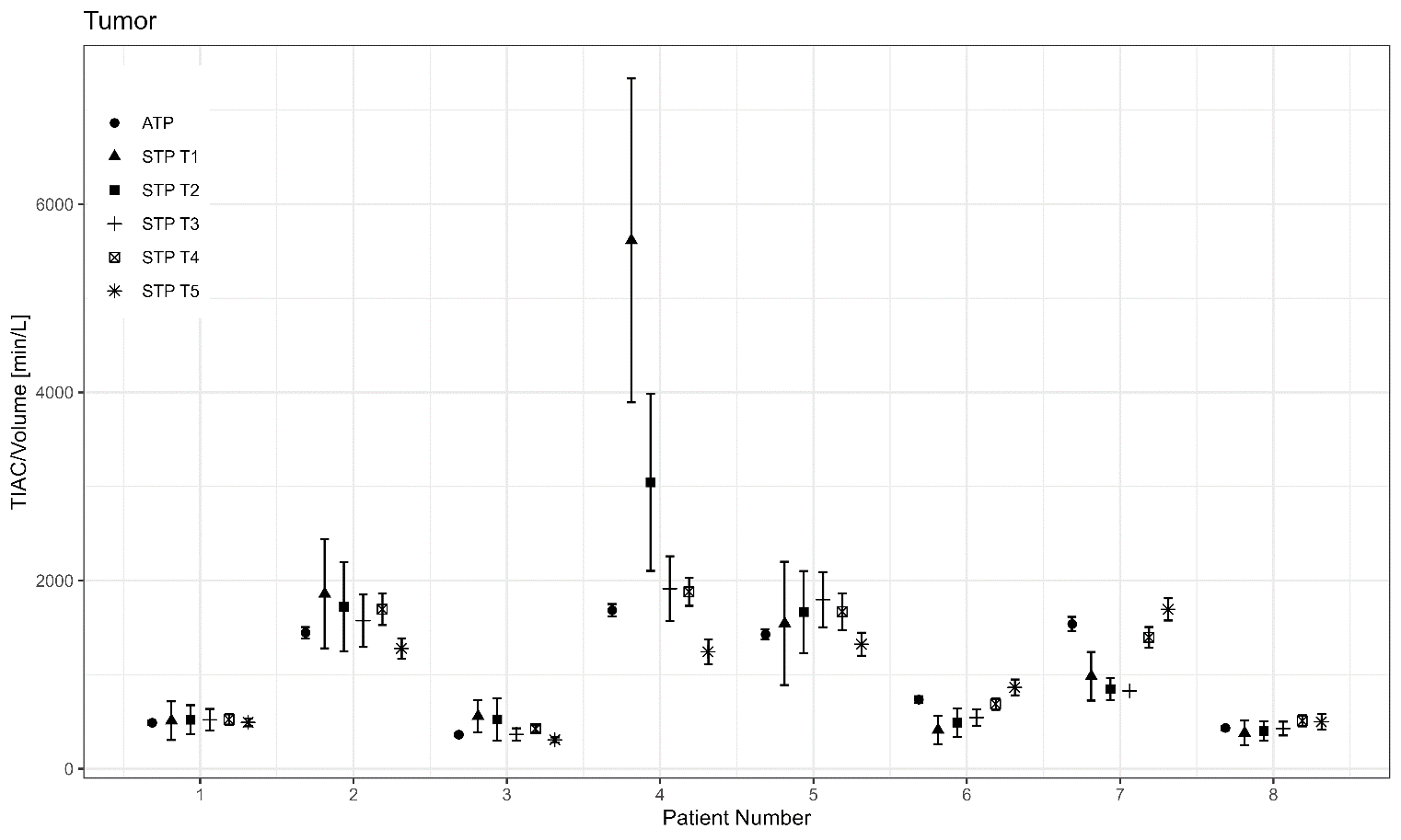


Figure S4. Time-integrated activity coefficient (TIAC) for serum, whole body, kidneys, spleen, liver, and tumour, obtained from all-time-point (ATP) and single-time-point (STP) imaging. The TIAC for tumours are presented in TIAC/volume. In STP fitting, blood serum data of the specific STP patient were excluded.

**REFERENCES**

1. Kletting P, Kull T, Maaß C, Malik N, Luster M, Beer AJ, et al. Optimized Peptide Amount and Activity for ^90^Y-Labeled DOTATATE Therapy. Journal of Nuclear Medicine. 2016;57:503-8. doi:10.2967/jnumed.115.164699.

2. Hardiansyah D, Riana A, Beer A, Glatting G. Single-time-point estimation of absorbed doses in PRRT using a non-linear mixed-effects model. Z Med Phys. 2023;33:70-81.

3. Antunes P, Ginj M, Zhang H, Waser B, Baum RP, Reubi JC, et al. Are radiogallium-labelled DOTA-conjugated somatostatin analogues superior to those labelled with other radiometals? Eur J Nucl Med Mol Imaging. 2007;34:982-93. doi:10.1007/s00259-006-0317-x.

4. Kimura H, Takeuchi H, Koshimoto Y, Arishima H, Uematsu H, Kawamura Y, et al. Perfusion imaging of meningioma by using continuous arterial spin-labeling: comparison with dynamic susceptibility-weighted contrast-enhanced MR images and histopathologic features. AJNR Am J Neuroradiol. 2006;27:85-93.

5. Guyennon A, Mihaila M, Palma J, Lombard-Bohas C, Chayvialle JA, Pilleul F. Perfusion characterization of liver metastases from endocrine tumors: Computed tomography perfusion. World J Radiol. 2010;2:449-54. doi:10.4329/wjr.v2.i11.449.

6. Gear JI, Cox MG, Gustafsson J, Gleisner KS, Murray I, Glatting G, et al. EANM practical guidance on uncertainty analysis for molecular radiotherapy absorbed dose calculations. European Journal of Nuclear Medicine and Molecular Imaging. 2018;45:2456-74. doi:10.1007/s00259-018-4136-7.
